# Metallomic analysis of brain tissues distinguishes between cases of dementia with Lewy bodies, Alzheimer’s disease, and Parkinson’s disease dementia

**DOI:** 10.3389/fnins.2024.1412356

**Published:** 2024-06-26

**Authors:** Melissa Scholefield, Stephanie J. Church, Jingshu Xu, Garth J. S. Cooper

**Affiliations:** ^1^Division of Cardiovascular Sciences, School of Medical Sciences, Faculty of Biology, Medicine and Health, The University of Manchester, Manchester Academic Health Science Centre, Manchester, United Kingdom; ^2^Faculty of Science, School of Biological Sciences, University of Auckland, Auckland, New Zealand

**Keywords:** dementia with Lewy bodies (DLB), Alzheimer’s disease (AD), Parkinson’s disease dementia (PDD), metallomics analysis, copper, human brain study, ICP-MS (inductively coupled plasma-mass spectrometry)

## Abstract

**Background:**

Dementia with Lewy bodies (DLB) can be difficult to distinguish from Alzheimer’s disease (AD) and Parkinson’s disease dementia (PDD) at different stages of its progression due to some overlaps in the clinical and neuropathological presentation of these conditions compared with DLB. Metallomic changes have already been observed in the AD and PDD brain—including widespread decreases in Cu levels and more localised alterations in Na, K, Mn, Fe, Zn, and Se. This study aimed to determine whether these metallomic changes appear in the DLB brain, and how the metallomic profile of the DLB brain appears in comparison to the AD and PDD brain.

**Methods:**

Brain tissues from ten regions of 20 DLB cases and 19 controls were obtained. The concentrations of Na, Mg, K, Ca, Zn, Fe, Mn, Cu, and Se were determined using inductively coupled plasma-mass spectrometry (ICP-MS). Case–control differences were evaluated using Mann–Whitney U tests. Results were compared with those previously obtained from AD and PDD brain tissue, and principal component analysis (PCA) plots were created to determine whether cerebral metallomic profiles could distinguish DLB from AD or PDD metallomic profiles.

**Results:**

Na was increased and Cu decreased in four and five DLB brain regions, respectively. More localised alterations in Mn, Ca, Fe, and Se were also identified. Despite similarities in Cu changes between all three diseases, PCA plots showed that DLB cases could be readily distinguished from AD cases using data from the middle temporal gyrus, primary visual cortex, and cingulate gyrus, whereas DLB and PDD cases could be clearly separated using data from the primary visual cortex alone.

**Conclusion:**

Despite shared alterations in Cu levels, the post-mortem DLB brain shows very few other similarities with the metallomic profile of the AD or PDD brain. These findings suggest that while Cu deficiencies appear common to all three conditions, metal alterations otherwise differ between DLB and PDD/AD. These findings can contribute to our understanding of the underlying pathogenesis of these three diseases; if these changes can be observed in the living human brain, they may also contribute to the differential diagnosis of DLB from AD and/or PDD.

## Background

Dementia with Lewy bodies (DLB) is a neurodegenerative disease characterised by progressive cognitive decline, followed by motor dysfunction of onset one year or more later; this contrasts with the similarly presenting Parkinson’s disease dementia (PDD), in which motor symptoms precede cognitive symptoms by at least one year (McKeith et al., 2017). Taken together, DLB and PDD comprise the Lewy body dementias (LBDs) and are distinguished only by the order of the onset of clinical symptoms.

Neuropathologically, the LBDs are also very similar, with both showing dopaminergic neuronal loss that is primarily localised within the substantia nigra pars compacta (SNpc), but which spreads throughout the brain as the disease progresses. This spread may follow a different regional pattern depending on the DLB subtype of the patient, with five subtypes defined by the McKeith criteria: olfactory bulb-only, amygdala-predominant, brainstem-predominant, limbic (transitional), and diffuse neocortical (McKeith et al., 2017). Of these, the diffuse neocortical and limbic (transitional) are the most common subtypes, with the former affecting the frontal, temporal, and parietal lobes and the latter primarily involving transitional regions such as the amygdala, transentorhinal cortex, and cingulate cortex. This differs to the α-synuclein Braak staging typically used to stage PDD (Braak et al., 2006). The loss of dopaminergic neurons is accompanied by the deposition of Lewy bodies, comprising proteinaceous deposits primarily composed of misfolded α-synuclein. The most notable difference in the neuropathological presentation of DLB and PDD is that in the former, there may be a more widespread cortical deposition of Lewy bodies; however, there is a high degree of overlap even in the neuropathology of the LBDs.

Adding to the difficulties of making a correct DLB diagnosis is that it can also present similarly to another dementia—Alzheimer’s disease (AD)—in several aspects, particularly in the earliest stages of the disease. Before the onset of motor symptoms or in the absence of hallucinations, DLB can look very similar to AD in the clinic. Some clinical differences can be used to differentiate DLB from AD, such as a higher prevalence of hallucinations, rapid eye-movement sleep behavior disorder (RBD), and GI symptoms such as constipation, but these symptoms cannot be used to provide a definitive differential diagnosis. Furthermore, as with PDD, DLB and AD share some neuropathological similarities; although deposits of misfolded tau and amyloid-β are the neuropathological hallmarks of AD, they are also observed in a high percentage of post-mortem DLB brains (Irwin et al., 2017), and α-synuclein inclusions can even be observed in many cases of AD (Twohig and Nielsen, 2019). Even further complicating matters, it is possible for individuals to present with mixed AD and LBD; as such, even at post-mortem, making the correct diagnosis can present significant challenges.

These challenges with making a correct diagnosis of DLB both pre-and post-mortem present several difficulties to both the medical and research communities. Patients who receive an incorrect diagnosis during their lifetime may receive treatments that not only do not address symptoms, but may even exacerbate their disease; for example, antidopaminergic medications such as neuroleptic antipsychotics are contraindicated in DLB, as DLB patients appear particularly sensitive to a condition known as neuroleptic malignant syndrome (NMS)—a condition which can worsen Parkinsonian motor dysfunction, depression, anxiety, and delusions (Hershey and Coleman-Jackson, 2019). The correct diagnosis of LBDs and mixed dementias is also essential for the suitable selection of study cohorts in research.

Such difficulties in distinguishing DLB from other conditions has led to the question of whether the underlying processes are similar between diseases or whether the differences that are observed in their clinical progression indicate differing mechanisms. For instance, changes in the metallomic profile of both the LBDs and AD have been reported; for example, widespread decreases in Cu have been observed throughout both the PD (Ayton et al., 2013; Davies et al., 2014; Genoud et al., 2017; Scholefield et al., 2021) and AD (Deibel et al., 1996; Loeffler et al., 1996; Akatsu et al., 2012; Xu et al., 2017) brain, as well as more localised alterations in Mn (Corrigan et al., 1993; Genoud et al., 2017; Xu et al., 2017; Scholefield et al., 2021), Se (Corrigan et al., 1993; Xu et al., 2017; Scholefield et al., 2021), Na (Vitvitsky et al., 2012; Xu et al., 2017; Scholefield et al., 2021), Zn (Corrigan et al., 1993; Deibel et al., 1996; Religa et al., 2006; Genoud et al., 2017; Xu et al., 2017; Scholefield et al., 2021), Ca (Xu et al., 2017), and Fe (Dexter et al., 1991; Deibel et al., 1996; Ayton et al., 2013; Raven et al., 2013; Szabo et al., 2016; Costa-Mallen et al., 2017; Genoud et al., 2017). Investigations into the metallomic profile of the DLB brain, however, are more scarce, although there have been limited reports of decreased Cu in the hippocampus (HP) (Akatsu et al., 2012) and frontal cortex (fCX) (Magaki et al., 2007). However, even in these limited data there are disagreements, with the latter study reporting no changes in HP Cu levels (Magaki et al., 2007). Studies have also reported no Cu changes in the DLB neocortex (nCX) (Graham et al., 2014) and amygdala (Akatsu et al., 2012), no Fe changes in the HP, amygdala (Akatsu et al., 2012), or nCX (Graham et al., 2014), as well as no changes in Mn or Zn levels (Akatsu et al., 2012).

This study aimed to create an extensive metallomic profile of the post-mortem DLB brain by quantifying the concentrations of eight essential metals and Se across ten brain regions. This profile was then compared with those of AD and PDD brains, obtained using the same methodologies in age-and sex-matched cohorts. These data were used first to characterise case–control differences in metals that may contribute to pathogenic mechanistic processes occurring within the DLB brain, and then to ascertain whether metals data from these three diseases can be used to distinguish DLB, PDD, and AD metallomic profiles at post-mortem.

## Results

### Cohort characteristics

Tissues were obtained from 10 regions from a total of 23 DLB cases and 20 controls. As not every case/control had tissue available for each region, *n* values for individual regions varied; these are shown in Table 1.

**Table 1:** Number of cases and controls per investigated region.

	HP	MED	MTG	CG	PVC	PONS	MCX	PUT	SN	CB
Controls	14	15	15	15	16	14	16	13	18	16
Cases	15	15	15	15	15	8	15	8	15	12

Cases and controls were matched as closely as possible for age at death, sex distribution, and post-mortem delay (PMD). In the overall cohort there were no significant differences between case–control age and sex, but cases did show a shorter average PMD than controls (14.7 vs. 18.4 h, respectively; *p* = 0.02; see Table 2); between cases and control groups, this small significant difference was present in the HP, 21, and SN, but not for regions (see Supplementary Table S2). As a previous analysis our lab conducted by our lab determined that PMD does not have a significant effect on cerebral metal concentrations (Scholefield et al., 2020), it was decided that these minor differences in PMD would not affect the suitability of the cohort for the current study.

**Table 2:** Overall cohort characteristics.

	Age at death (years)	Sex (% male)	PMD (hours)
Controls (*n* = 20)	73.6 (65–85)	57.9	18.4 (8.1–29.1)
Cases (*n* = 23)	74.1 (65–85)	60.0	14.7 (8.0–22.6)

This table shows data for age and PMD as mean (range). PMD, post-mortem delay.

### DLB metallomics

The levels of eight essential metals (Na, Mg, K, Ca, Mn, Fe, Cu, and Zn) and Se were measured in ten regions (HP, MED, pons, MCX, CG, CB, MTG, SN, PVC, and PUT) of DLB and control brains using ICP-MS.

**Figure 1:**
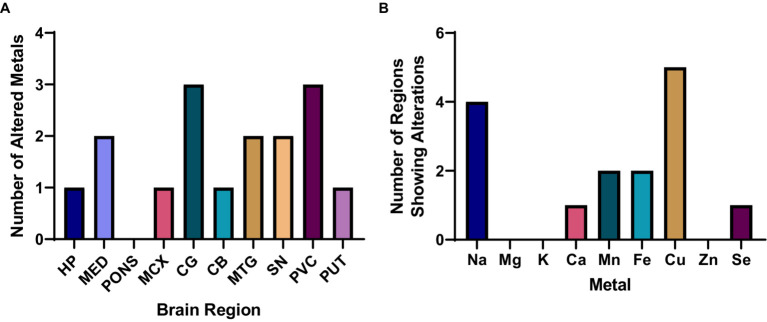
Overall metallomic profile of DLB brains. **(A)** Number of changes observed per region; **(B)** Number of changes observed per metal. HP, Hippocampus; MED, Medulla oblongata; MCX, Motor cortex; CG, Cingulate gyrus; CB, Cerebellum; MTG, Middle temporal gyrus; SN, Substantia nigra; PVC, Primary visual cortex; PUT, Putamen.

The highest number of metallic alterations were observed in the CG and PVC (three each), followed by the MTG and SN (two changes), one change in the HP, MCX, CB, and PUT, and no significant alterations in the pons (see Figure 1A). As such, the pattern of metallomic changes did not appear to reflect the level of neuropathology usually observed throughout the DLB brain, in which areas such as the SN, HP, and MCX usually show the highest levels of neurodegeneration and amyloid and/or α-synuclein pathology.

In terms of which metals were found to be altered in the DLB brain, Cu and Na were significantly altered in the highest number of regions—at five and four regions, respectively—followed by significant alterations in Mn and Fe in two regions, and Ca and Se changes in one region each (see Figure 1B). No significant case–control differences in Mg, K, or Zn were observed in any region. As such, Cu and Na changes were the most widespread alterations observed, with more localised alterations in Ca, Mn, Fe, and Se.

**Figure 2:**
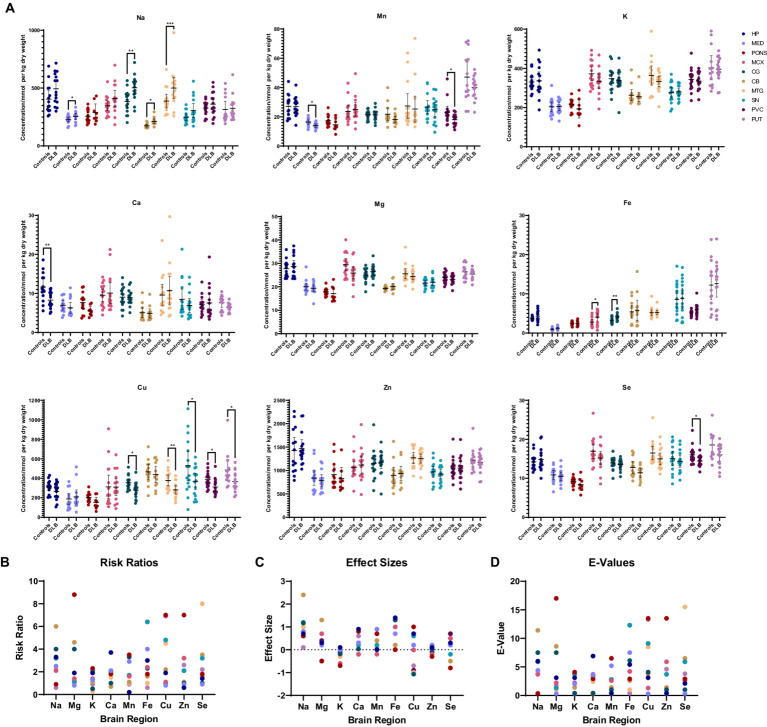
**(A)** Graphs show concentrations of analytes in DLB cases and controls across ten regions of the brain. Case–control difference determined by nonparametric Mann–Whitney U test. * *p* < 0.05, ** *p* < 0.01, *** *p* < 0.001. **(B–D)** Graphs show risk ratios, effect sizes, and *E*-values for each metal in each region of the brain.

Table 3 and Figure 2A show individual metallic alterations in individual brain regions; risk ratios, effect sizes, and *E*-values are shown in Table 3 and Figures 2B-D. Na was found to be increased in the MED (227.9 v 254.0 mmol/kg, respectively; *p* = 0.04), CG (383.7 vs. 502.3 mmol/kg, respectively; *p* = 0.004), MTG (387.1 vs. 499.2 mmol/kg, respectively; *p* = 0.02), and CB (172.0 vs. 211.9 mmol/kg, respectively; *p* = 0.014) of DLB cases in comparison to controls. Ca was decreased in the HP (11.6 vs. 8.3 mmol/kg, respectively; *p* = 0.009). Mn was decreased in the MED (16.4 vs. 1.5 μmol/kg, respectively; *p* = 0.0107) and PVC (23.1 vs. 18.0 μmol/kg, respectively; *p* = 0.03). Fe was increased in the MCX (2.8 vs. 4.1 mmol/kg, respectively; *p* = 0.03) and CG (3.2 vs. 4.1 mmol/kg, respectively; *p* = 0.004). Cu was decreased in the CG (355.6 vs. 279.3 μmol/kg; *p* = 0.02), MTG (378.6 vs. 281.4 μmol/kg, respectively; *p* = 0.007), SN (530.5 vs. 367.2 μmol/kg, respectively; *p* = 0.01004), PVC (386.6 vs. 311.3 μmol/kg, respectively; *p* = 0.012), and PUT (486.0 vs. 366.2 μmol/kg, respectively; *p* = 0.04). Se was decreased in the PVC (16.0 vs. 14.4 μmol/kg, respectively; *p* = 0.04).

**Table 3:** Regional alterations in DLB brain metals.

(A) Metal alterations in the HP								
HP	Controls	Cases	*p*-value	*S*-value	*E*-value	Risk Ratio (RR)	RR CI *E*-values	Effect size
Na	403.5 ± 74.1 (275.2–531.9)	493.1 ± 75.1 (357.5–628.6)	0.08	3.6	6.0	3.3 (0.8–13.1)	1.0	0.7
Mg	27.7 ± 2.1 (24.1–31.4)	28.7 ± 2.6 (26.1–31.3)	0.8	0.3	3.1	1.9 (0.4–8.6)	1.0	0.3
K	330.9 ± 26.9 (284.3–377.5)	336.5 ± 45.3 (254.6–418.3)	0.9	0.2	3.1	1.9 (0.4–8.6)	1.0	0.1
Ca	11.6 ± 2.1 (8.0–35.1)	8.3 ± 1.2 (6.1–10.4)	0.009	6.8	6.9	3.7 (0.95–14.7)	1.0	0.9
Mn	27.1 ± 4.6 (19.1–35.1)	26.9 ± 3.6 (20.4–33.4)	0.9	0.2	0.4	0.2 (0.03–1.8)	1.0	0.0
Fe	3.7 ± 0.2 (3.3–4.1)	4.3 ± 0.8 (2.9–5.7)	0.6	0.7	5.4	3.0 (0.7–12.4)	1.0	1.4
Cu	312.4 ± 39.0 (244.9–379.8)	266.6 ± 47.9 (180.1–353.1)	0.3	1.7	3.1	1.9 (0.6–6.1)	1.0	0.7
Zn	1426.7 ± 279.2 (943.1–1910.3)	1469.4 ± 189.0 (1128.2–1810.6)	0.7	0.5	0.4	0.6 (0.1–6.1)	1.0	0.1
Se	14.2 ± 1.2 (12.1–16.3)	14.8 ± 1.6 (11.8–17.7)	0.6	0.7	2.1	1.4 (0.3–7.2)	1.0	0.3

(B) Metal alterations in the MED								
MED	Controls	Cases	*p*-value	*S*-value	*E*-value	Risk Ratio (RR)	RR CI *E*-values	Effect size
Na	227.9 ± 20.0 (193.2–262.6)	254.0 ± 25.2 (210.5–297.7)	0.04	4.6	4.4	2.5 (1.02–6.1)	1.2	0.8
Mg	20.0 ± 1.6 (17.2–22.8)	19.4 ± 1.4 (16.9–21.8)	0.9	0.2	0.3	0.8 (0.2–3.0)	1.0	0.2
K	203.3 ± 24.5 (159.14–247.48)	205.0 ± 19.9 (170.5–239.5)	0.5	1.0	2.0	1.3 (0.4–4.0)	1.0	0.0
Ca	6.9 ± 1.2 (5.0–8.9)	6.3 ± 1.4 (3.9–8.7)	0.2	2.3	3.6	2.1 (0.8–5.2)	1.0	0.3
Mn	16.4 ± 1.5 (13.8–19.0)	13.9 ± 1.1 (12.0–15.8)	0.0107	6.5	5.2	2.9 (0.8–7.2)	1.0	0.9
Fe	1.0 ± 0.2 (0.7–1.3)	1.2 ± 0.2 (0.8–1.6)	0.09	3.5	7.5	4.0 (1.1–17.3)	1.4	0.7
Cu	188.4 ± 55.6 (88.0–288.8)	209.3 ± 72.0 (84.6–334.1)	0.5	1.0	0.3	0.8 (0.2–3.0)	1.0	0.2
Zn	837.5 ± 55.6 (558.3–1116.7)	784.3 ± 126.7 (565.0–1003.7)	0.5	1.0	0.3	0.9 (0.2–2.6)	1.0	0.2
Se	10.9 ± 1.1 (8.9–12.9)	10.5 ± 1.1 (8.7–12.4)	0.5	1.0	0.3	0.9 (0.2–2.6)	1.0	0.2

(C) Metal alterations in the pons								
PONS	Controls	Cases	*p*-value	*S*-value	*E*-value	Risk Ratio (RR)	RR CI *E*-values	Effect size
Na	256.3 ± 57.0 (223.3–289.2)	288.1 ± 88.9 (213.9–362.4)	0.50	1.4	0.3	0.9 (0.2–3.8)	1.0	0.6
Mg	17.9 ± 1.6 (17.0–18.8)	17.1 ± 3.3 (14.3–19.9)	0.4	0.9	17.0	8.8 (1.2–62.4)	1.8	−0.5
K	214.7 ± 30.4 (197.1–232.2)	193.1 ± 55.4 (146.8–239.4)	0.3	1.6	4.1	2.3 (0.7–7.9)	1.0	−0.7
Ca	7.6 ± 2.6 (6.1–9.1)	5.6 ± 1.3 (4.5–6.6)	0.07	5.5	2.9	1.8 (0.5–6.7)	1.0	0.8
Mn	17.2 ± 3.8 (15.1–19.4)	14.5 ± 4.2 (11.0–18.0)	0.1	2.7	6.5	3.5 (0.8–15.0)	1.0	0.7
Fe	2.4 ± 0.5 (2.2–2.7)	2.5 ± 0.7 (1.8–3.1)	0.90	0.0	2.9	1.8 (0.3–10.1)	1.0	0.0
Cu	203.4 ± 49.7 (174.6–232.1)	151.9 ± 65.9 (96.8–207.0)	0.1	3.6	13.5	7.0 (0.9–52.3)	1.0	1.0
Zn	912.4 ± 286.9 (746.7–1078.0)	831.8 ± 279.6 (598.0–1065.5)	0.5	0.9	13.5	7.0 (0.4–154.8)	1.0	−0.3
Se	9.2 ± 1.0 (8.6–9.8)	8.4 ± 1.8 (6.9–9.9)	0.2	1.8	2.9	1.8 (0.5–6.7)	1.0	−0.8

(D) Metal alterations in the MCX								
MCX	Controls	Cases	*p*-value	*S*-value	*E*-value	Risk Ratio (RR)	RR CI *E*-values	Effect size
Na	346.5 ± 79.6 (304.1–388.9)	408.6 ± 129.4 (337.0–480.3)	0.1	3.3	3.7	2.1 (0.5–10.0)	1.0	0.8
Mg	29.5 ± 4.9 (26.9–32.1)	25.9 ± 4.6 (23.4–29.5)	0.06	4.1	2.2	1.4 (0.4–5.3)	1.0	0.7
K	372.2 ± 66.4 (336.8–407.6)	334.3 ± 66.1 (297.7–370.9)	0.3	1.7	2.2	1.4 (0.4–5.3)	1.0	−0.6
Ca	9.5 ± 2.9 (8.0–11.1)	10.1 ± 4.9 (7.4–12.8)	0.9	0.2	3.7	2.1 (0.5–10.0)	1.0	−0.2
Mn	23.6 ± 7.3 (19.7–33.7)	25.0 ± 8.7 (20.2–29.8)	0.4	1.3	2.6	1.6 (0.3–8.3)	1.0	−0.2
Fe	2.8 ± 1.3 (2.1–3.5)	4.1 ± 1.8 (3.1–5.1)	0.03	5.1	4.2	2.4 (0.9–6.2)	1.0	1.0
Cu	313.3 ± 223.7 (194.1–432.6)	310.5 ± 155.1 (224.6–396.4)	0.5	1.0	1.3	1.1 (0.02–50.4)	1.0	−0.01
Zn	1064.2 ± 254.7 (928.5–1200.0)	1118.4 ± 346.3 (926.6–1310.1)	0.7	0.5	5.9	3.2 (0.2–4.2)	1.0	−0.2
Se	17.0 ± 3.4 (15.1–18.8)	15.2 ± 3.1 (13.5–16.9)	0.2	2.3	2.6	1.6 (0.2–4.2)	1.0	0.5

(E) Metal alterations in the CG								
CG	Controls	Cases	*p*-value	*S*-value	*E*-value	Risk Ratio (RR)	RR CI *E*-values	Effect size
Na	383.7 ± 101.8 (327.4–440.1)	502.3 ± 106.3 (443.4–561.2)	0.004	8.0	7.5	4.0 (1.01–15.8)	1.1	1.17
Mg	25.0 ± 3.0 (23.4–26.7)	26.5 ± 2.9 (24.9–28.1)	0.3	1.7	7.5	4.0 (0.5–31.7)	1.0	−0.49
K	347.4 ± 60.4 (314.0–380.9)	336.9 ± 59.0 (304.2–369.5)	0.8	0.3	0.4	0.5 (0.1–4.9)	1.0	−0.18
Ca	9.0 ± 2.8 (7.4–10.7)	8.9 ± 2.1 (7.7–10.1)	0.9	0.2	0.4	1.0 (0.2–4.2)	1.0	0.03
Mn	21.3 ± 3.0 (19.7–22.9)	21.3 ± 3.9 (19.1–23.4)	1.0	0.0	1.0	3.3 (1.1–9.8)	1.0	0.02
Fe	3.2 ± 0.7 (2.8–3.6)	4.1 ± 0.9 (3.6–4.6)	0.004	8.0	6.1	2.3 (0.7–7.4)	1.5	1.31
Cu	355.6 ± 71.0 (316.3–394)	279.3 ± 83.4 (233.2–325.5)	0.02	6.0	4.1	1.0 (0.1–14.6)	1.0	−1.07
Zn	1146.3 ± 321.4 (968.3–1324.3)	1179.5 ± 254.2 (1038.7–1320.2)	0.5	1.0	1.0	1.0 (0.2–4.2)	1.0	−0.10
Se	13.9 ± 1.6 (13.0–14.8)	13.6 ± 1.4 (12.8–14.3)	0.5	1.0	1.0	1.0 (0.2–4.2)	1.0	0.21

(F) Metal alterations in the SN								
SN	Controls	Cases	*p*-value	*S*-value	*E*-value	Risk Ratio (RR)	RR CI *E*-values	Effect size
Na	240.8 ± 52.3 (213.9–267.7)	305.0 ± 109.5 (244.4–365.7)	1.0	0.0	5.9	3.2 (0.8–13.5)	1.0	1.2
Mg	21.5 ± 1.9 (20.5–22.4)	21.9 ± 2.4 (20.6–23.3)	0.9	0.2	1.3	1.1 (0.2–6.6)	1.0	0.3
K	279.3 ± 51.0 (254.0–304.7)	280.0 ± 34.6 (260.9–299.2)	0.8	0.3	3.7	2.1 (0.2–21.2)	1.0	0.0
Ca	8.0 ± 4.7 (5.5–10.4)	6.9 ± 3.2 (5.1–8.7)	0.6	0.7	3.4	2.0 (0.4–9.3)	1.0	0.2
Mn	26.2 ± 7.7 (22.2–30.1)	24.7 ± 8.7 (19.9–29.6)	0.8	0.3	2.8	1.7 (0.3–8.8)	1.0	0.2
Fe	8.8 ± 4.3 (6.7–11.0)	8.9 ± 4.7 (6.2–11.5)	1	0.0	12.3	6.4 (0.9–47.1)	1.0	0.0
Cu	530.5 ± 272.3 (395.1–665.9)	367.2 ± 149.5 (284.5–450.0)	0.1004	3.3	9.1	4.8 (1.2–18.7)	1.8	0.6
Zn	952.0 ± 211.8 (843.1–1060.9)	926.0 ± 180.6 (826.0–1026.0)	0.9	0.2	3.7	2.1 (0.2–21.2)	1.0	−0.1
Se	14.9 ± 3.4 (13.2–16.6)	14.4 ± 2.8 (12.8–15.9)	0.5	1.0	5.9	3.2 (0.8–13.5)	1.0	−0.2

(G) Metal alterations in the CG								
CB	Controls	Cases	*p*-value	*S*-value	*E*-value	Risk Ratio (RR)	RR CI *E*-values	Effect size
Na	172.0 ± 10.1 (155.3–188.8)	211.9 ± 21.0 (178.9–232.9)	0.0135	13.3	11.4	6.0 (1.6–21.6)	2.7	2.4
Mg	19.1 ± 0.5 (18.3–19.9)	20.1 ± 1.3 (18.2–22.1)	0.09	3.5	8.6	4.6 (0.6–33.4)	1.0	1.3
K	271.5 ± 41.7 (202.5–340.5)	255.4 ± 22.7 (219.6–291.2)	0.9	0.2	1.4	1.1 (0.02–50.4)	1.0	−0.2
Ca	5.5 ± 1.3 (3.4–7.6)	4.9 ± 1.2 (3.0–6.7)	0.5	1.0	0.3	0.7 (0.1–3.6)	1.0	0.3
Mn	21.2 ± 4.5 (14.2–28.2)	18.1 ± 2.1 (14.7–21.4)	0.1	3.3	1.4	1.1 (0.1–15.6)	0.0	0.4
Fe	5.4 ± 1.6 (2.7–8.1)	5.9 ± 2.3 (2.3–9.6)	0.8	0.3	2.6	1.6 (0.5–9.8)	1.0	0.2
Cu	455.0 ± 59.2 (356.9–553.0)	439.6 ± 83.5 (308.2–570.9)	0.7	0.5	3.8	2.2 (0.5–9.8)	1.0	0.2
Zn	903.8 ± 152.5 (605.2–1202.4)	937.8 ± 152.5 (697.8–1177.9)	0.5	1.0	1.4	1.1 (0.2–6.5)	1.0	0.1
Se	13.1 ± 1.9 (9.9–16.3)	11.4 ± 1.2 (9.6–13.3)	0.4	1.3	6.5	3.5 (0.03–2.2)	1.0	−0.5

(H) Metal alterations in the MTG								
MTG	Controls	Cases	*p*-value	*S*-value	*E*-value	Risk Ratio (RR)	RR CI *E*-values	Effect size
Na	387.1 ± 59.2 (280.2–494.0)	499.2 ± 92.2 (332.7–665.6)	0.02	5.6	4.1	2.3 (0.7–7.4)	1.0	1.0
Mg	25.6 ± 2.1 (21.7–29.4)	24.3 ± 1.3 (21.9–26.7)	1	1.0	1.0	1.0 (0.1–14.6)	1.0	0.3
K	363.3 ± 48.9 (274.9–451.7)	333.3 ± 26.9 (284.7–381.9)	0.4	1.3	0.4	0.3 (0.04–2.9)	1.0	−0.3
Ca	9.6 ± 2.8 (4.6–14.5)	10.7 ± 3.6 (4.1–17.2)	0.7	0.5	2.4	1.5 (0.3–7.7)	1.0	−0.2
Mn	27.4 ± 8.4 (12.2–42.5)	25.3 ± 8.9 (9.3–41.3)	0.4	1.3	1.0	1.0 (0.02–47.4)	1.0	0.1
Fe	5.2 ± 0.8 (3.9–6.6)	5.2 ± 0.5 (4.4–6.1)	0.7	0.5	1.0	1.0 (0.1–15.6)	1.0	0.0
Cu	378.6 ± 55.3 (278.7–478.4)	281.4 ± 42.9 (203.9–358.8)	0.007	7.2	8.5	4.5 (1.2–17.4)	1.0	−1.0
Zn	1272.0 ± 120.6 (1054.3–1489.7)	1255.3 ± 96.4 (1081.2–1429.4)	0.8	0.3	1.0	1.0 (0.2–6.2)	1.0	0.1
Se	16.5 ± 1.7 (13.5–19.6)	15.0 ± 1.5 (12.3–17.7)	0.1	3.3	15.5	8.0 (0.5–153.8)	1.0	0.5

(I) Metal alterations in the PVC								
PVC	Controls	Cases	*p*-value	*S*-value	*E*-value	Risk Ratio (RR)	RR CI *E*-values	Effect size
Na	326.2 ± 61.7 (293.3–359.1)	360.6 ± 105.1 (302.4–418.8)	0.5	1.0	5.9	3.2 (0.8–13.5)	1.0	0.6
Mg	23.9 ± 2.2 (22.8–25.1)	23.1 ± 2.1 (22.0–24.3)	0.4	1.3	1.3	1.1 (0.2–6.6)	1.0	0.4
K	343.7 ± 56.8 (313.4–374.0)	335.4 ± 44.7 (310.7–360.2)	0.7	0.5	3.7	2.1 (0.2–21.2)	1.0	−0.1
Ca	7.1 ± 2.3 (5.9–8.3)	7.6 ± 4.3 (5.2–9.9)	0.7	0.5	3.4	2.0 (0.4–9.3)	1.0	−0.2
Mn	23.1 ± 7.7 (19.0–27.2)	18.0 ± 3.9 (15.8–20.1)	0.03	5.1	2.8	1.7 (0.3–8.8)	1.0	0.7
Fe	5.1 ± 0.6 (4.8–5.5)	5.9 ± 1.6 (5.1–6.8)	0.1	3.6	12.3	6.4 (0.9–47.1)	1.0	1.3
Cu	386.6 ± 80.8 (343.5–429.6)	311.3 ± 82.4 (265.7–356.9)	0.012	6.4	9.1	4.8 (1.4–67.0)	2.1	−0.9
Zn	1054.8 ± 220.6 (937.2–1172.3)	1048.6 ± 255.4 (907.2–1190.1)	0.9	0.2	3.7	2.1 (0.2–21.2)	1.0	0.03
Se	16.0 ± 2.2 (14.8–17.2)	14.4 ± 1.4 (13.7–15.2)	0.04	4.6	5.9	3.2 (0.8–13.7)	1.0	0.7

(J) Metal alterations in the PUT								
PUT	Controls	Cases	*p*-value	*S*-value	*E*-value	Risk ratio (RR)	RR CI *E*-values	Effect size
Na	314.5 ± 117.5 (243.5–385.5)	324.7 ± 104.1 (267.1–382.4)	0.5	1.0	0.4	0.6 (0.1–3.4)	1.0	0.1
Mg	26.4 ± 3.3 (24.3–28.5)	25.4 ± 2.1 (24.3–26.6)	0.5	1.0	1.7	1.2 (0.2–6.1)	1.0	0.3
K	402.9 ± 101.9 (341.3–464.5)	395.9 ± 63.1 (360.9–430.8)	0.8	0.3	0.3	0.9 (0.1–5.3)	1.0	−0.1
Ca	7.6 ± 1.9 (6.4–8.8)	6.4 ± 1.0 (5.8–7.0)	0.1	4.1	0.4	0.7 (0.2–2.4)	1.0	0.6
Mn	47.1 ± 17.3 (36.6–57.6)	40.0 ± 7.9 (35.6–44.4)	0.50	1.0	0.3	0.9 (0.1–2.9)	1.0	0.4
Fe	12.2 ± 6.1 (8.5–15.9)	13.7 ± 7.1 (9.8–17.7)	0.70	0.5	0.4	0.6 (0.1–2.9)	1.0	0.2
Cu	486.0 ± 178.6 (378.0–595.9)	366.2 ± 104.4 (308.4–424.0)	0.04	4.6	13.3	6.9 (0.5–133.8)	1.0	−0.7
Zn	1222.7 ± 246.1 (1074.0–1371.4)	1171.9 ± 215.4 (1052.6–1291.1)	1.0	0.0	4.6	2.6 (0.3–22.1)	1.0	0.2
Se	18.6 ± 3.5 (16.5–20.7)	16.0 ± 2.9 (14.4–17.6)	0.07	3.8	3.8	2.2 (0.5–9.3)	1.0	0.7

Analyte concentrations are shown as mean ± standard deviation (confidence interval). Case–control differences were determined using nonparametric Mann–Whitney U tests. Na, Mg, K, Ca, and Fe values are in mmol/kg; Mn, Cu, Zn, and Se values are in μmol/kg. Risk ratios are shown as mean (confidence interval). RR, Risk ratio; CI, Confidence interval.

In order to determine the potential effects of covariates such as age, sex, tau Braak stage, and PMD, a two-way multivariate analysis of variance (MANCOVA) analysis was carried out for each brain region—firstly to determine any overall effects of these variates, and then to determine any effects on individual metals in different brain regions. Regarding the overall effects of covariates in different brain regions, tau Braak stage had a statistically significant effect (*p* < 0.05) in the CB (*F* = 0.01; *p* = 0.006; see Supplementary Table S3) and CG (*F* = 6.7; *p* = 0.03), and age had a statistically significant effect in the MED (*F* = 10.3; *p* = 0.04). Regarding effects on individual metals in different brain regions, tau Braak stage had a significant, positive effect on Na in multiple regions including the pons (*F* = 6.5; *p* = 0.02; see Supplementary Table S4 and Supplementary Figure S4), MTG (*F* = 8.0; *p* = 0.01), MCX (*F* = 18.5; *p* < 0.001), CB (*F* = 10.1; *p* = 0.005), and SN (*F* = 5.0; *p* = 0.04), as well as a positive effect on Fe in the CG (*F* = 18.0; *p* < 0.001) and a negative effect on Cu in the PVC (*F* = 9.3; *p* = 0.009). Sex showed a significant effect on some metals, with Se levels being lower in women in the MTG (*F* = 5.6; *p* = 0.03) and CG (*F* = 10.9; *p* = 0.006), Mg and K being higher in women in the CB (*F* = 6.4; *p* = 0.02 and *F* = 5.5; *p* = 0.03, respectively), and Mn being higher in women in the SN (*F* = 6.3; *p* = 0.02). Age had a positive effect on Se levels in the PVC (*F* = 9.1, *p* = 0.009).

**Figure 3:**
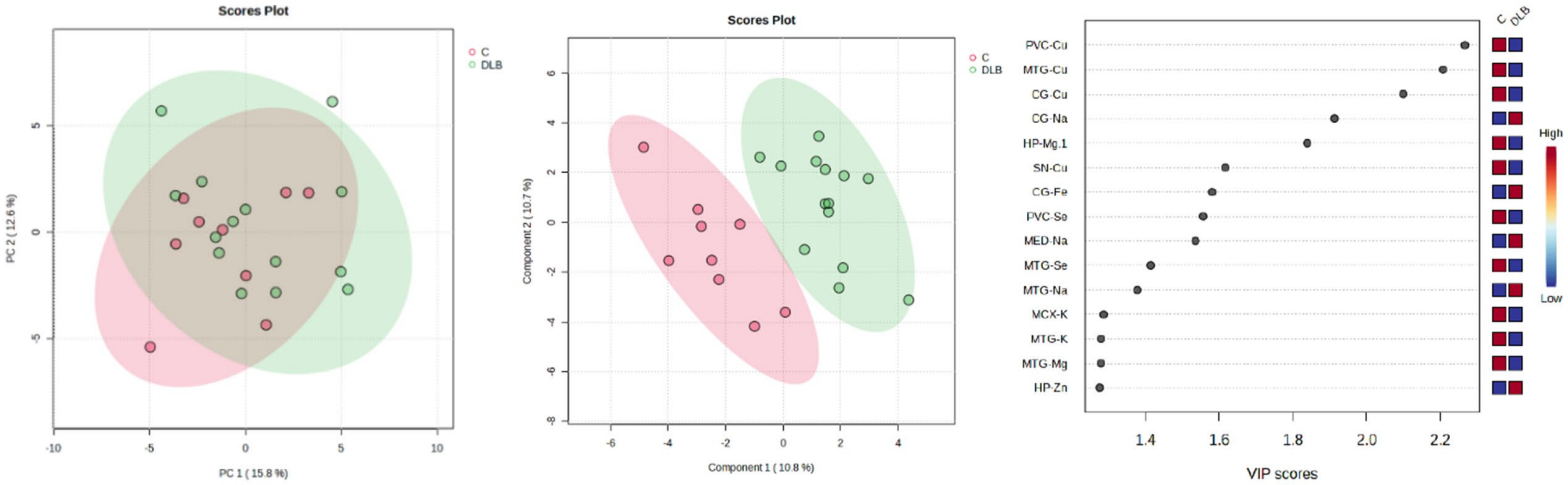
PCA and PLS-DA separation of DLB cases and controls. Image shows PCA (left) and PLS-DA (middle) plots of VIP scores of DLB cases and controls in green and red, respectively, with VIP scores on the right.

PCA analyses were performed to determine whether overall or regional metallomic changes could distinguish DLB from control samples, but no separation was observed (see Figure 3A). PLS-DA plots achieved separation when using data for all comparable regions (the CB data were excluded from this analysis as the donors used for this region differed from those used for other regions; see Figure 3B); the VIP scores indicated that Cu changes contributed most to this separation, in accordance with the number of case–control alterations observed (see Figure 3C). It was investigated whether any changes were seen if DLB cases could be separated based on the presence of AD pathology, but no separation was observed (data not shown).

### DLB vs. AD vs. PDD comparisons

ICP-MS analyses of AD (Xu et al., 2017) and PDD (Scholefield et al., 2021) brains have previously been carried out using the same methodologies as those employed in this study. In AD, the CB, CG, HP, MCX, MTG, and PVC were investigated, as well as the entorhinal cortex, which was not investigated in the current study. In PDD, the CB, CG, HP, MCX, MED, MTG, pons, PVC, and SN were investigated. As such, six regions can be compared between AD and DLB, nine between DLB and PDD, and six between all three dementias.

**Figure 4:**
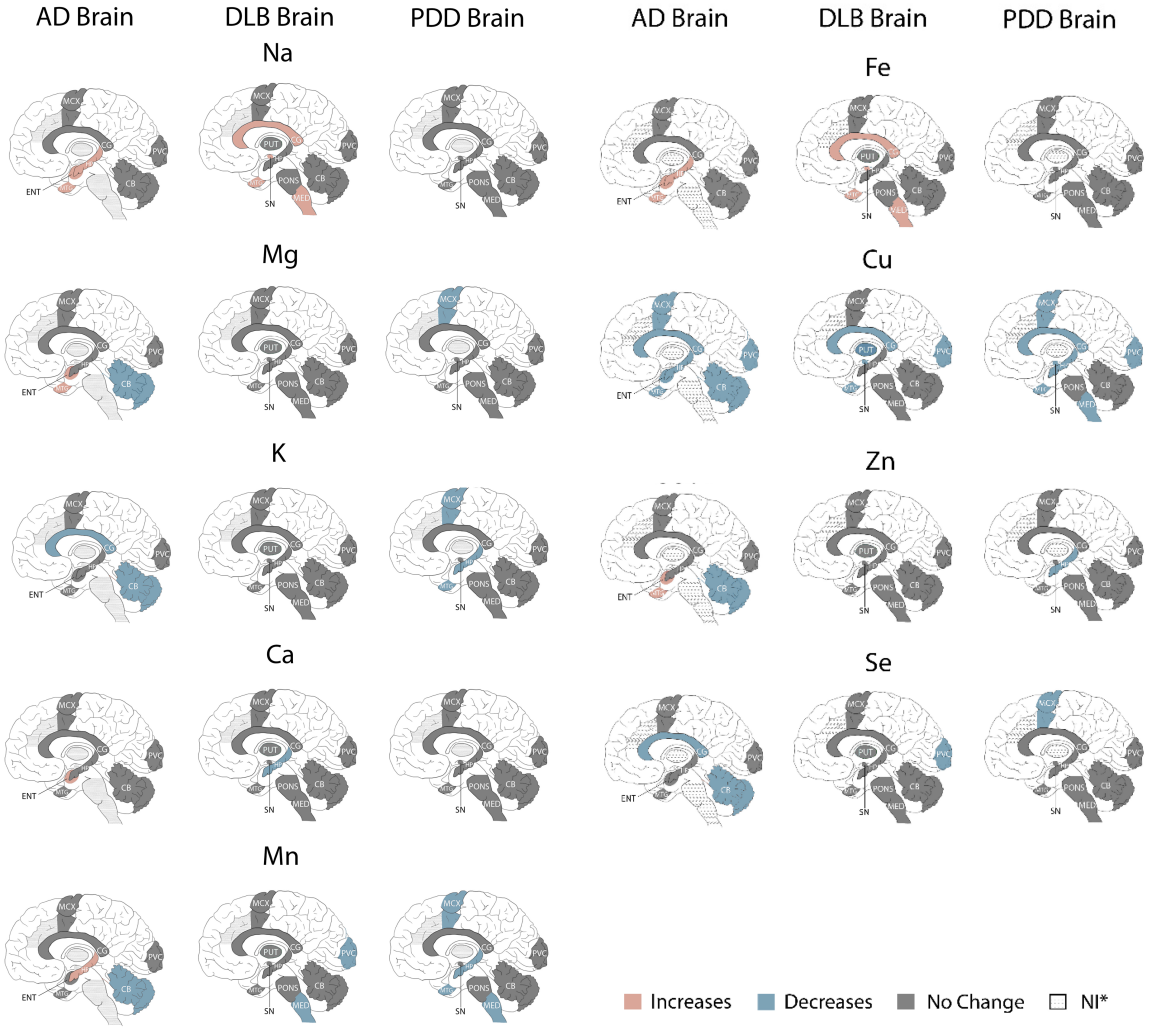
Metallomic profiles of AD, DLB, and PDD brains. Image shows significant Case–control differences in AD, DLB, and PDD brains, respectively, according to the brain regions affected. Red shading indicates increased concentrations compared to controls, blue shading indicates decreases, and grey shading indicates no significant differences. *Dotted shading indicates that region was not investigated.

Directly comparing the significant case–control differences observed in each condition, the most striking similarity is that of the Cu findings, with Cu showing widespread decreases in all three diseases; this includes all seven regions investigated in AD and the CG, HP MCX, MED, MTG, PCX, and SN in PDD (see Figure 4). As such, Cu was decreased in the CG, MTG, and PVC in all three dementias, with shared Cu decreases also observed in the SN in both DLB and PDD; this region was not investigated in AD. In contrast, the hippocampal Cu decreases observed in both AD and PDD were not seen in the DLB brain.

With the exception of Cu changes, there was only one other alteration shared between DLB and AD and/or PDD brains—decreased Mn in both the DLB and PDD MED. The localised changes in Na, Ca, Fe, and Se seen in the DLB brain were not comparable across the same regions in either AD or PDD. Overall, there appeared to be fewer metallic changes in the DLB compared to the AD brain (15 alterations overall in DLB vs. 25 in the AD brain), but only slightly fewer than in the PDD brain (17 alterations overall), with none of the changes in Zn, K, or Mg that were seen in AD and/or PDD. As such, there appears to be a lower degree of metallomic involvement in the DLB brain compared to the AD brain and a similar degree as seen in the PDD brain.

### Disease separation by PCA and PLS-DA

To identify whether the observed metallomic changes could be used to distinguish DLB cases from those with AD or PDD, PCA and PLS-DA analyses were carried out. VIP scores from PLS-DA plots were used to identify the regions most heavily contributing to any separation observed; regions contributing to a lesser degree were gradually removed until the minimum number of regions needed to distinguish DLB from AD and/or PDD was determined. As the samples used to identify metal levels in the CB were mostly different to those used for the investigation of other brain regions in DLB, these were not included in multi-disease PCA and PLS-DA analyses.

**Figure 5:**
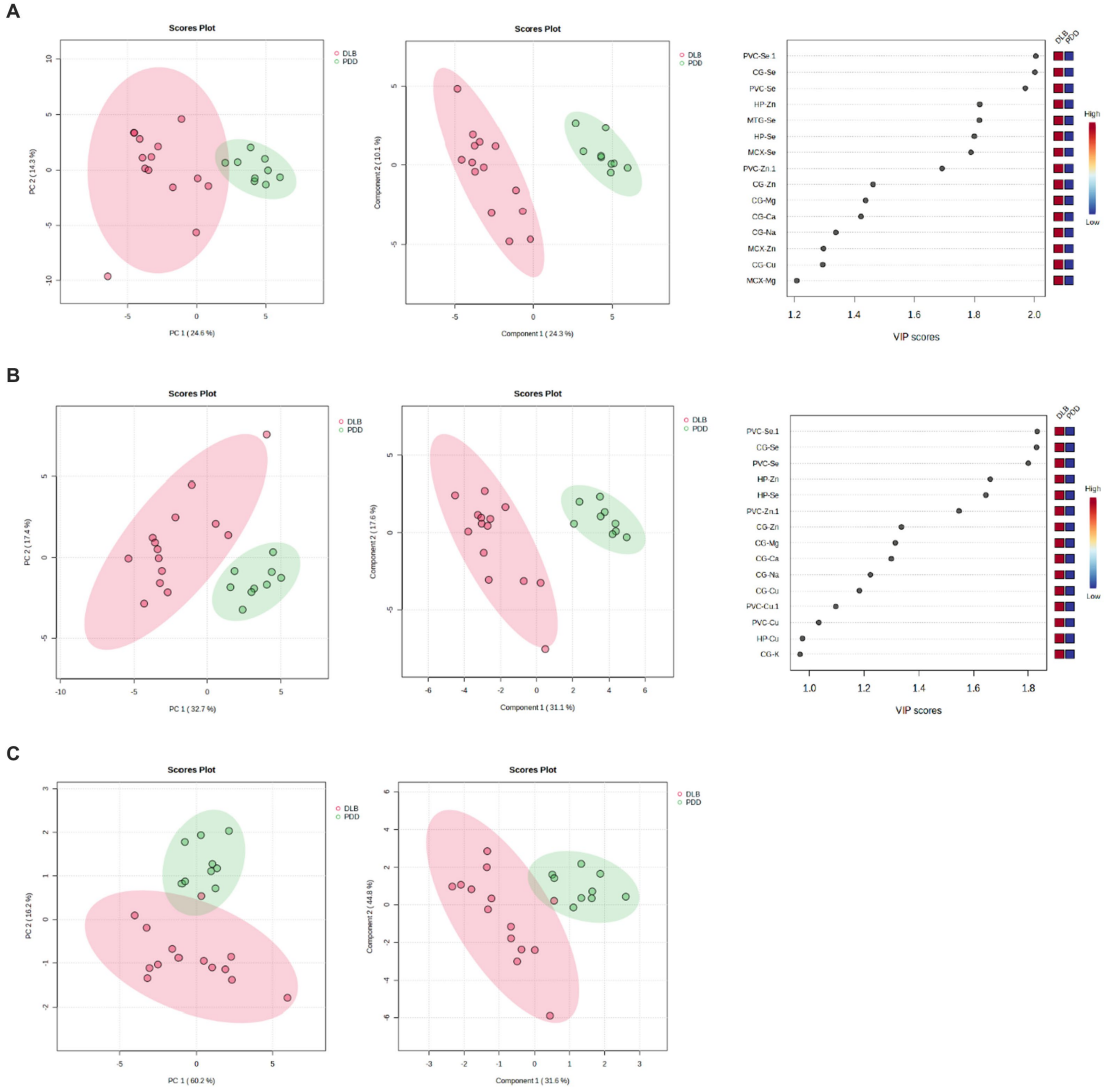
PCA and PLS-DA separation of DLB and PDD cases. Separation based on data obtained from **(A)** CG, HP, MTG, MCX, MED, SN, and PVC; **(B)** PVC, CG, and HP; and (C) PVC only. Plots are ordered as follows: PCA plot, PLS-DA plot, VIP scores of PLS-DA plot. DLB cases are shown in red and PDD cases in green.

Separation of DLB and PDD brains was achieved using the data obtained from CG, HP, MTG, MCX, MED, SN and PVC samples (see Figure 5A); VIP scores indicated that the PVC, CG, and HP Se and Zn alterations contributed most to this separation. The best separation of DLB and PDD samples was achieved using only these three regions (see Figure 5B). However, separation was still possible even with only the use of PVC data (see Figure 5C). As such, it may be possible to distinguish PDD from DLB using metallomic data from the PVC alone.

**Figure 6:**
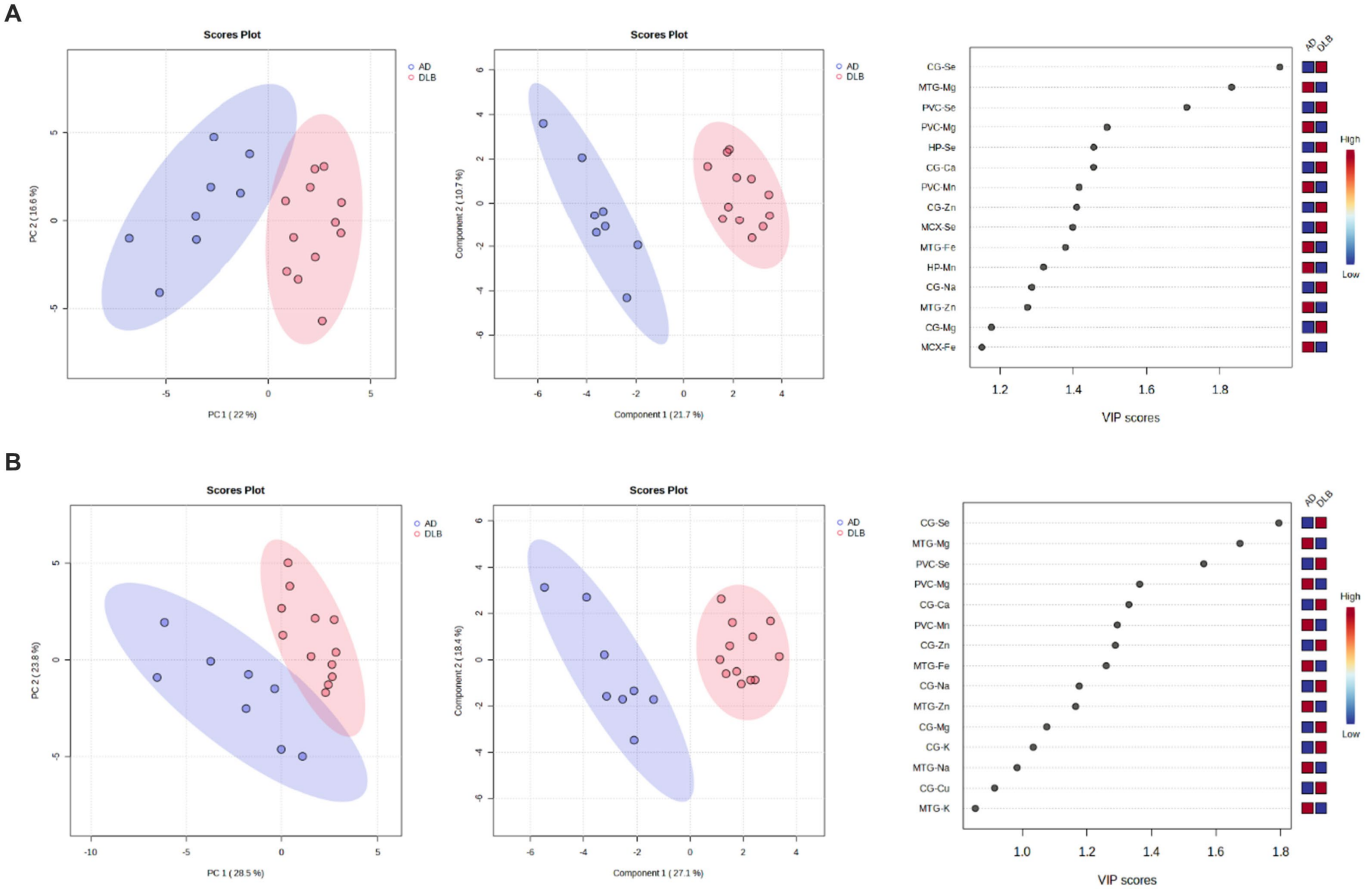
6PCA and PLS-DA separation of DLB and AD cases. Separation based on data obtained from the **(A)** CG, HP, MTG, PVC, and MCX; **(B)** CG, MTG, and PVC. Plots are ordered as follows: PCA plot, PLS-DA plot, VIP scores of PLS-DA plot. DLB cases are shown in red and AD cases in blue. Plots are ordered as follows: PCA plot, PLS-DA plot, VIP scores of PLS-DA plot. DLB cases are shown in red, PDD cases in green, and AD cases in blue.

Clear separation of DLB and AD samples was achieved using the data obtained from CG, HP, MTG, PVC, and MCX samples (see Figure 6A); VIP scores indicated that the CG, MTG, and PVC Se and Mg data contributed most to this separation. Good separation was still achieved using only these three regions (see Figure 6B). The VIP scores for this separation indicated that the CG and MTG contributed most strongly, but removal of PVC data resulted in a loss of separation between DLB and AD brains. As such, the MTG, PVC, and CG metallomic data were all required as a minimum to distinguish DLB from AD brain samples.

**Figure 7:**
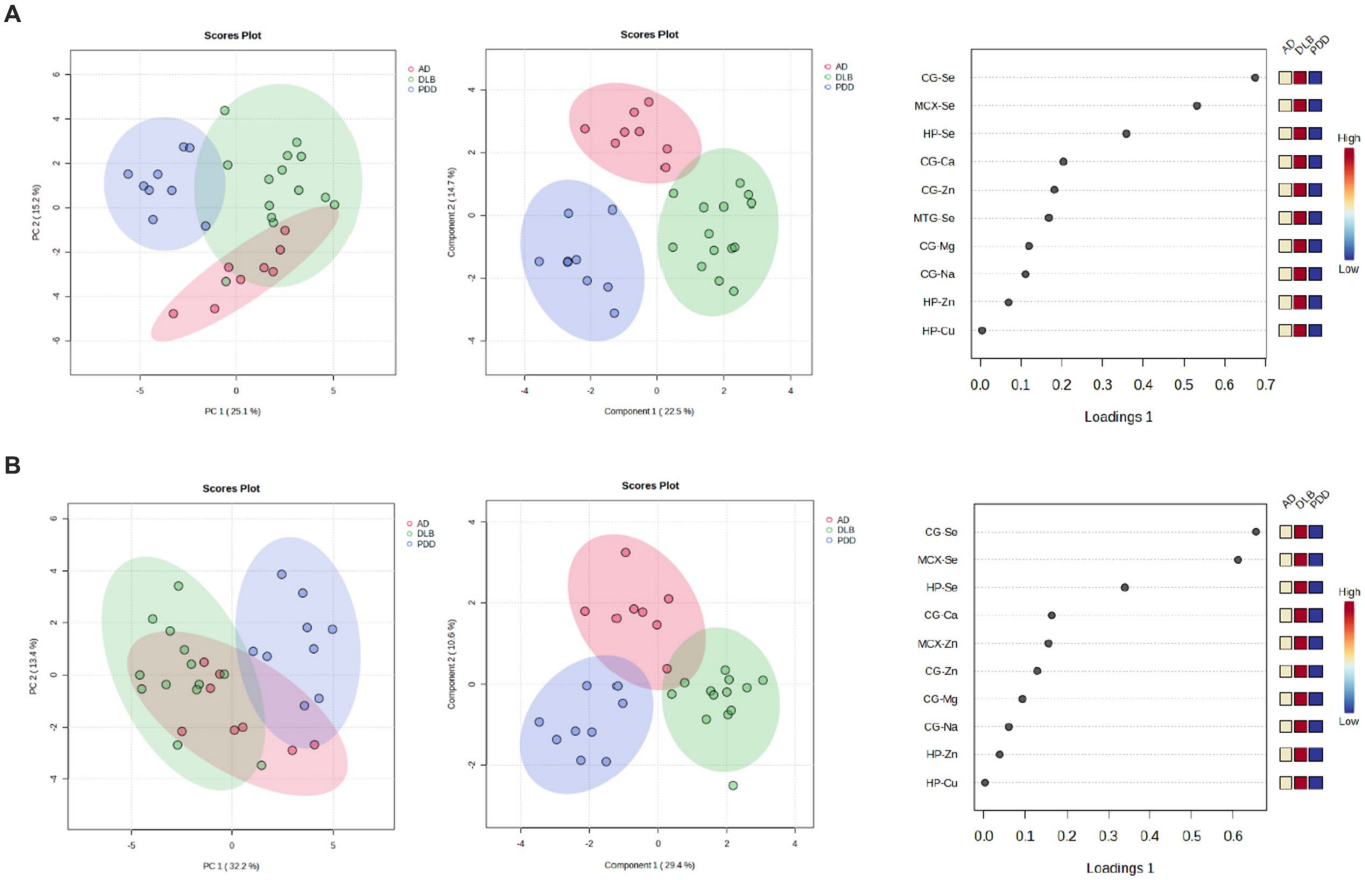
7PCA and PLS-DA separation of AD, DLB, and PDD cases. Separation based on data taken from the **(A)** CG, HP, MTG, and MCX; **(B)** MTG, MCX, and HP. Plots are ordered as follows: PCA plot, PLS-DA plot, VIP scores of PLS-DA plot. DLB cases are shown in red, PDD cases in green, and AD cases in blue.

When looking at all three diseases simultaneously, a good separation of DLB and PDD samples was possible using CG, HP, MTG, and MCX metallomic data (see Figure 7A); there was, however, some overlap between AD and DLB samples. VIP scores indicated that the MTG, MCX, and HP contributed the most to this separation; however, separation was lost with the removal of the CG data (see Figure 7B).

## Discussion

ICP-MS analysis of DLB samples revealed fairly widespread Cu decreases and Na increases in five and four out of ten investigated regions, respectively, as well as more localised changes in Mn, Ca, Fe, and Se. No alterations were found in K, Mg, or Zn. Using these metallomic data, DLB samples could be distinguished from AD and PDD samples using PCA, with only data from the PVC being required to separate DLB samples from PDD samples, and data from the MTG, PVC, and CG separating DLB from AD brains. As such, diminished Cu levels may contribute to pathogenic mechanisms in the DLB brain—as also previously observed in PDD and AD brains—but the relative metallomic profiles of each of these three dementias may be able to distinguish each from the others. This is of particular importance, as at present it can be difficult to correctly diagnose DLB using pre-mortem clinical presentation alone.

### Cu, Mn, Fe, and se

There have been limited reports on Cu levels in the DLB brain in the past, including some reports of decreases in localised regions such as the HP (Akatsu et al., 2012) and fC (Magaki et al., 2007), but also other reports of no changes in the HP (Magaki et al., 2007), as well as the amygdala (Akatsu et al., 2012) and NCx (Graham et al., 2014). As such, investigations of DLB cerebral Cu have been fairly limited up to this point and have not always agreed. In the present study, hippocampal Cu was not found to be significantly altered, but Cu levels were instead observed to be diminished in the CG, MTG, SN, PVC, and PUT—including not only regions with high typical levels of neurodegeneration in DLB such as the SN, but also those with relatively lowers levels such as the PVC. This suggests fairly widespread alterations in Cu throughout the DLB brain that are not directly correlated to dopaminergic neuronal loss or Lewy body deposition. Cu was found to have negative correlation with tau Braak stage in the PVC, but not in any other investigated region—there were, however, only a few samples in the current study with higher tau Braak stages of 5 or 6, which may have influenced the results of the MANCOVA analysis, and so this result requires further investigation in a larger study.

Cu is an essential metal in the human brain, acting as a cofactor for many key metabolic enzymes—in particular, antioxidants such as superoxide dismutase 1 (SOD1) and ceruloplasmin, as well as proteins involved in energy production such as cytochrome c oxidase, one of the components of the mitochondrial electron transport chain (Ruiz et al., 2021). As such, Cu is involved in a range of essential metabolic processes in the brain from antioxidation to ATP production—also having roles in neuronal signalling, myelin formation and maintenance, and gene regulation (An et al., 2022).

Although there is an abundance of evidence indicating the presence of oxidative stress and mitochondrial dysfunction in both AD and PDD, evidence concerning the DLB brain is much more limited; however, there have been reports of oxidative damage in the DLB brain even at early stages of the disease—in particular, involving increases in the levels of advanced glycation end products (AGEs) and their receptors as well as in the lipoxidation of α-synuclein in the fCX (Dalfo and Ferrer, 2008), even prior to alterations in α-synuclein aggregation. In addition, decreases in mitochondrial O_2_ uptake and complex I activity have also been observed in the DLB fCX—considered to be a result of mitochondrial oxidative damage (Navarro et al., 2009)—as well as increases in neuronal RNA (Nunomura et al., 2002) and protein, lipid, and DNA oxidation (Lyras et al., 1998) throughout the DLB cortex. Transcriptomic analysis of post-mortem anterior cingulate and dorsolateral prefrontal cortex tissue from LBD patients has also indicated gene expression alterations that indicate the presence of oxidative stress and mitochondrial dysfunction (Rajkumar et al., 2020). At present, these reports appear to be limited to the DLB cortex, but the Cu alterations observed in the present study may indicate more widespread oxidative stress and mitochondrial dysfunction throughout the DLB brain.

Although alterations in Mn, Fe, and Se were much more localised than Cu changes—affecting the PVC, MCX and CG, and PVC, respectively—they are also involved in mitochondrial function and oxidative processes (Brenneisen et al., 2005; Li and Yang, 2018; Galaris et al., 2019). As such, the dysregulation of these metals may compound any disruption already caused to these pathways by Cu dysregulation. For example, Mn is a SOD2 cofactor, whereas Se is a cofactor for glutathione peroxidases and thioredoxin reductases. SOD2 has been reported to be a target of lipoxidation in incidental LBD cortex, as well as alpha-synuclein itself, reflecting the presence of oxidative stress (Dalfo et al., 2005). There have been reports of increased GPX1 levels in DLB microglia, with affected microglia showing higher levels of hypertrophy and neuronal contacts (Power and Blumbergs, 2009). Additionally, the same group also observed that non-selenium GPX levels are increased in the DLB cortical astrocytes (Power et al., 2002). Decreases in reduced glutathione levels have also been observed in the SN of presymptomatic LBD patients, without any concurrent changes in Fe, Cu, Mn, or Zn levels—although Se levels were not investigated (Dexter et al., 1994). Such increases in antioxidative factors may reflect a response to increased levels of oxidative stress in the DLB brain, but without sufficient levels of Se, this response may be attenuated in the DLB PVC, compounding the effects of Mn and Cu loss in this region.

Previous studies have found no changes in Fe levels in the DLB frontal cortex and HP (Magaki et al., 2007) or neocortex (Graham et al., 2014), but the current authors were unable to find any previous investigations of the MCX or CG. Increased Fe levels are also associated with oxidative stress, largely through the production of peroxynitrite and hydroxyl radicals via the Fenton reaction (Winterbourn, 1995). Furthermore, Fe-induced oxidative stress has been observed to increased α-synuclein protein levels in HEK293 cells (Koukouraki and Doxakis, 2016), as well as Fe itself being capable of directly binding to α-synuclein (Peng et al., 2010). The phosphorylation of α-synuclein, which is considered to be an essential step in the formation of Lewy bodies (Anderson et al., 2006), increases the protein’s affinity for ferrous ions (Lu et al., 2011), and such binding could lead to increased α-synuclein translation through disruption of the protein binding to its iron-responsive element in the 5′-untranslated region (Lu et al., 2011). Fe binding has also been proposed to increase α-synuclein aggregation, with α-synuclein in turn being observed to cause Fe accumulation in cell models (Levin et al., 2011; Ortega et al., 2016). As such, Fe and α-synuclein may create a vicious cycle in which each induces further accumulation of the other, leading to further negative effects such as oxidative stress and protein toxicity.

Taken together, dysregulation of Cu, Mn, Fe, and Se suggest the widespread presence of oxidative stress and mitochondrial dysfunction in the DLB brain, which may be compounded in regions with more than one dysregulated analyte.

### Na

Na was found to be increased in four regions of the DLB brain: the MED, CG, CB, and MTG. Na ions are a crucial component of cellular homeostasis, regulating action potential generation and conduction alongside K ions via Na^+^–K^+^ ATPase. Although K alterations were not observed here in the DLB brain, changes in Na^+^ ion concentrations could be sufficient to upset the delicate balance required for the regulation of action potentials. Na levels appeared to show a positive correlation with tau Braak stage in several investigated regions, including the pons, MTG, CG, MCX, CB, and SN, indicating a potential relationship between Na accumulation and tau pathology; however, these results should be interpreted with caution due to the lower number of samples with higher tau Braak stages and require further validation in larger cohorts.

Neither Na nor K levels in DLB brains have received much attention in past works, with only a single study reporting no changes in the DLB parietal cortex compared to controls (Graham et al., 2015). Likewise, although ATPase activity has been reported to be disturbed in AD (de Lores Arnaiz and Ordieres, 2014), the authors could find no similar studies investigating the DLB brain. The Na/K pump requires substantial levels of ATP to properly function—up to a fifth of available cellular ATP (Harris et al., 2012). The primary process by which ATP is produced in cells is glucose-dependent; as such, the glucose hypometabolism that is observed in the DLB brain may lead to subsequent disturbances in Na levels (Ishii et al., 1998; Higuchi et al., 2000; Andersen et al., 2023). Considering the lack of data available on Na levels and Na^+^-K^+^ ATPase function in the DLB brain, they may represent a feature of interest in future investigations.

### Ca

Ca was only observed to be altered in one region of the DLB brain, being decreased in the HP. However, like Na, Ca also has important roles in cellular homeostasis and cell signalling, being a major intracellular messenger (Kawamoto et al., 2012; Santulli and Marks, 2015). Therefore, alterations in Ca levels may have several negative consequences, including disruptions in protein folding, transcriptional regulation, and mitochondrial function. Ca has also been observed to bind to α-synuclein and increase its aggregation and oligomerisation (Nielsen et al., 2001). However, in a study by Gomez–Tortosa et al., it was observed that human cortical neurons expressing calcium-binding proteins taken from DLB patients did not develop Lewy bodies (Gomez-Tortosa et al., 2001). As such, whether Ca may have toxic effects in DLB via its interactions with α-synuclein or protective effects via its interactions with calcium-binding proteins on neurons requires further investigation.

### Distinguishing DLB from AD and PDD

Due to the neuropathological and clinical similarities between DLB and AD and PDD, diagnosis can be difficult both during an individual’s lifetime and at post-mortem. This can lead to unfortunate consequences, including slow or unsuitable treatment for the individual whilst alive, and difficulties in accurately identifying candidates for research studies both pre-and post-mortem.

Using PCA, it was possible to distinguish DLB cases from both AD and PDD cases in the current study; using data from three regions—the MTG, PVC, and CG—DLB cases could be separated from AD cases, and using only data from the PVC, DLB and PDD cases could also be separated. This highlights that although there are some similarities across all three diseases—most notably, widespread Cu decreases—there are also sufficient differences to distinguish one disease from another at post-mortem.

These differences may indicate differing neuropathological processes between DLB and AD/PDD; for example, the diminished Mn and Se levels in the DLB PVC—not present in AD or PDD—may contribute to DLB’s differing clinical progression. Hallucinations may occur in any of these diseases, but are particularly prevalent in DLB, being considered a core clinical feature according to the DLB consortium criteria (McKeith et al., 2017). As such, the PVC is a region of particular interest in DLB, particularly with regards to its differential diagnosis and for determining the differing mechanisms that contribute to its particular clinical presentation. PVC hypometabolism, as assessed using fluorodeoxyglucose (FDG)-positron emission tomography (PET), is already considered a supportive indicator for DLB diagnosis, and has also been suggested for use in differentiating DLB from AD (Fujishiro et al., 2012); however, whether this could also support the differential diagnosis of DLB from PDD has not yet been investigated to the authors’ knowledge. Although hallucinations are also often reported in PDD, they are more likely to follow the administration of dopaminergic therapy and less likely to occur spontaneously than in DLB patient (Jellinger and Korczyn, 2018). Some studies have shown reduced blood flow and hypometabolism within the DLB PVC, accompanied by indicators of reduced GABAergic activity, despite a lack of α-synuclein or neurofibrillary tangle pathology (Khundakar et al., 2016); such hypometabolism has also been observed to correlate with clinical features of DLB, regardless of the presence of cognitive decline. Together, current and previous findings within the DLB PVC suggest that this region is of particular interest for future research, particularly in the context of the differential diagnosis of DLB and the determination of cerebral alterations that lead to hallucinations in this disease.

The CG is also a region of interest in DLB due to the frequent presence of the cingulate island sign, in which there is occipital hypometabolism observable in the DLB brain by FDG-PET, but with relative sparing of the posterior cingulate cortex (Imabayashi et al., 2016). However, it should be noted that in the present study, tissue was taken from the anterior CG. Regardless, the anterior CG plays important roles in the consolidation of emotionally coded memories, and alterations in this region may contribute to the presence of dementia symptoms in DLB. The anterior CG has been observed to have higher levels of α-synuclein and amyloid-β burden than the posterior CG in DLB brains, which may contribute to the relative preservation of metabolism in the latte (Patterson et al., 2019).

The MTG plays roles both in language and semantic memory processing and visual perception and sensory integration; as such, changes in this brain region may contribute to both visual hallucinations and cognitive symptoms in DLB patients. Despite this, alterations to the MTG, and to the wider medial temporal lobe (MTL) as a whole, appear to be less prevalent than in AD. In terms of metallomic involvement, changes in the AD MTG appear quite extensive, including increases in Na, Mg, Zn, and Fe, as well as decreases in Cu, whereas the DLB MTG shows only increases in Na and Cu decreases. Previous imaging studies have observed that MTL atrophy occurs both in AD and DLB, but that it is more frequent in the former (Barber et al., 1999)—being able to distinguish AD from DLB and vascular cognitive impairment with a sensitivity and specificity of 91 and 94%, respectively (Burton et al., 2009). It has even been suggested that in pure DLB, without the presence of any AD pathology, MTL atrophy is so rare as to be a possible mitigating factor against a diagnosis of DLB (Lippa et al., 1998).

Taken together, the use of CG, MTG, and PVC data to distinguish DLB from AD seems to be in line with previous imaging study data. As such, the differences in metallomic findings observed here between the two may represent important mechanistic factors contributing to the differing clinical progression of DLB and AD—particularly with regards to the frequency of hallucinations in DLB with respect to changes in the PVC, and differing levels of cognitive decline in AD and DLB with respect to MTG and CG alterations. The use of the PVC to distinguish between DLB and PDD, however, appears to have received little attention to date; as such, this region should be of particular interest in future studies aiming to distinguish between the pathological or clinical progression of DLB and PDD.

### Strengths of weaknesses of the current study

To the authors’ knowledge, this study represents the most thorough characterisation of the metallomic profile of the DLB brain, analysing the levels of eight metals and Se across ten different regions with varying levels of neurodegeneration simultaneously. As most previous studies have focused on only a few metals across one or two heavily affected regions such as the SN or HP, many of the findings observed here have not previously been described. Sample numbers, although still small, are at least comparable if not higher than those of previous studies (Magaki et al., 2007; Akatsu et al., 2012; Graham et al., 2014, 2015), and were based on previous AD and PDD analyses in which nine cases vs. nine controls were sufficient to observe significant Case–control differences using this study’s methods (Xu et al., 2017; Scholefield et al., 2021). In this study we strove to increase the sample size to further strengthen the statistical power of the data in comparison to these previous studies. However, further validation in larger cohorts would still be of use, particularly for the investigation of any potential effects of covariates such as age, sex, and tau Braak stage; although such an analysis was performed here, it was limited by the small number of samples in many groups (e.g., one two samples each for tau Braak stage 5 and 6), and so must be interpreted with caution.

Another limiting factor may be the lack of available information on the individual donors forming the study cohort. Efforts were made to obtain as much information as possible, including age at death, sex, PMD, race, clinical brain diagnosis, DLB type (where applicable), α-synuclein and tau Braak stage, and comorbidities; this information is included in Supplementary Material A. However, this information was not available for all individuals, and other information such as cause of death, age at diagnosis, duration of disease, and medication history was not available. Efforts were made to minimise any effects from cohort variables such as age, sex, and PMD by matching of cases and controls, as well as keeping tau and α-synuclein Braak stage at <2 and 0 for controls, respectively. Despite this, disease progression can be incredibly varied, and both cases and controls presented with a variety of comorbidities—all of which could have unknown effects on cerebral metal levels. This does, however, reflect the real-life presentation of DLB in medical settings. Another limitation of this study is the lack of racial and ethnic diversity, with only one non-White donor in the current cohort. It is important to recognise that the recruitment of non-White tissue donors needs to improve, in order to investigate the reasons for observed differences in the presentation (Kurasz et al., 2020) and diagnosis (Kurasz et al., 2022; Bayram et al., 2023) of LBDs that have been noted in different racial/ethnic groups.

It is important to emphasise that as this study was an investigation of post-mortem tissues, it cannot draw conclusions on the disease stage at which identified changes occurred in the brain; as such, further studies either of the living brain using *in vivo* methods (e.g., MRI can be used to image some metals such as Fe *in vivo*) or peripheral studies of the plasma or serum are required to determine when such changes occur and whether they may have utility in the clinic as either biomarkers or potential therapeutic targets. The primary contribution of this manuscript is the determination of metallic changes that occur in the DLB brain and how these changes are similar to or differ from AD and PDD, and so identifying potential analytes of interest for further investigation—particularly in studies investigating the pathogenic mechanisms of these diseases.

## Conclusion

Widespread Cu decreases are present in the DLB brain, as previously observed in AD and PDD. More localised alterations in Na, Ca, Mn, Fe, and Se are also present in the DLB brain. Widespread Cu deficiencies appear common to DLB, AD, and PDD; however, the DLB brain otherwise shares few metallic alterations with either AD or PDD, suggesting that the underlying metallic dysfunction occurring in the brains of individuals with this disease differs to that seen in either AD or PDD. This suggests that despite the many clinical and neuropathological similarities between these conditions, the underlying pathogenesis may differ markedly.

## Materials and methods

### Reagents

Except where otherwise stated, all reagents were obtained from Sigma-Aldrich (UK).

### Acquisition of human brain tissues from DLB cases and controls

Brain tissues from 23 DLB cases and 20 controls were obtained from the NIH NeuroBioBank. Tissues from 10 different regions were identified and dissected by a consultant neuropathologist; these were the cerebellum (CB), substantia nigra (SN), motor cortex (MCX; Brodmann area 4), middle frontal gyrus (MFG; Brodmann area 46), middle temporal gyrus (MTG; Brodmann area 21), primary visual cortex (PVC; Brodmann area 17), cingulate gyrus (CG; Brodmann area 24), hippocampus (HP; Brodmann area 35), putamen (PUT), and the pons. As tissues for some of these regions was not available for some cases, actual *n* values for each region ranged from 8 cases v 14 controls (Pons) to 15 cases v 18 controls (SN).

### Ethics approval and consent to participate

All donors (and/or their families, when applicable) gave informed consent for the donation of the brain tissues used in this study. The collection of tissues was approved by the Auckland Human Participants Ethics Committee, the NIH NeuroBioBank, and MRC brain bank network; the study was reviewed, approved, and carried out in compliance with the approval made by Manchester REC (09/H0906/52 + 5).

Consent for the use of donated tissues for research and publication was collected by the UCLA Brain Bank and Harvard Brain Tissue Resource Center (DLB cohort), the ICL Brain Bank (PDD cohort), and the NZ Neurological Foundation Douglas Human Brain Bank (AD cohort).

### Diagnosis and severity of human cases

All DLB cases received a clinical diagnosis of DLB during their lifetime, as recorded in their medical notes. Upon collection of donated brain samples, neuropathologists at the Sepulveda and Harvard brain banks examined the donated brain tissues for pathological hallmarks of neurodegenerative disease and gave a neuropathological diagnosis of LBD to cases; details of DLB subtype, according to the McKeith criteria (McKeith et al., 2017) (limbic, diffuse, cortical, etc.), are provided in Supplementary Table S1, were made available in neuropathology reports. Some cases had comorbid Alzheimer’s neuropathology, as also indicated in Supplementary Table S1; Braak tau scores have been provided. Controls were not diagnosed with any evidence of dementia during their lifetime and did not show sufficient levels of neuropathology to receive a post-mortem diagnosis of any dementia; all controls had an α-synuclein Braak stage of 0 and a tau Braak stage of ≤2. Medical reports provided further characterisation of cases and controls, including data detailing comorbidities (see Supplementary Table S1).

### Tissue dissection

Brain tissue was stored at −80°C until analysis. On the experimental day, tissues were thawed slightly on ice to allow dissection. Tissues were sectioned into 50 mg (± 5%) wet weight for ICP–MS using a metal-free ceramic scalpel and placed into ‘Safe-Lok’ microfuge tubes (Eppendorf AG; Hamburg, Germany). The use of a ceramic scalpel prevented contamination from trace metals during sectioning.

### ICP-MS

Freshly dissected samples were briefly centrifuged before being dried for six hours to a constant weight (approximately 10 mg; individual sample wet and dry weights are provided in Supplementary Material B) using a Savant Speedvac™ (Thermo Fisher Scientific, Massachusetts, USA). Nitric acid digestion of samples was then performed in a heat block, along with digestion blanks containing nitric acid alone (see Supplementary Material B). Following digestion, samples were refrigerated overnight at 4°C before undergoing ICP-MS analysis with a 7,700x ICP-MS spectrometer (Agilent, Santa Clara, USA) equipped with a MicroMist nebulizer and Scott double-pass spray chamber (Glass Expansion, Melbourne, Australia), and nickel sample and skimmer cones. Samples were separated into batches of either one or two regions, with multi-element calibration using calibration standard dilutions (see Supplementary Material B for all raw data and values for blanks and standard curves). All elements were standardised against scandium, with the exception of zinc and selenium which were standardised against germanium. All regions were run in triplicate.

The Manufacturer’s recommendations were followed for selection of operation mode, integration times, and internal standard assignments. Samples were introduced to the instrument using an integrated autosampler (Agilent, Santa Clara, USA). The concentrations of eight essential metals (Na, Mg, K, Ca, Mn, Fe, Cu, and Zn) and the metalloid Se were determined. All elements were analysed using helium as the collision gas; selenium was analysed in high-energy helium mode (10 mL/min helium) due to its potential state as a polyatomic ion, and all other elements were analysed using standard helium mode (5·0 mL/min helium). Results were excluded from analysis where the highest blank value for any given analyte during a run was ≥15% of that of the lowest sample value.

### ICP-MS data analysis

Case–control age, sex, and PMD were compared using the non-parametric Mann–Whitney U test. Mean metal values [±standard deviation (SD) and with 95% confidence intervals (CI)] were calculated, normalised to the sample’s dry weight, and differences between cases and controls determined by Mann–Whitney U tests due to the small sample size. Shannon diversity indices (S-values also termed surprisal scores) were also calculated by taking the negative base 2 log of the *p*-value. Confidence intervals were calculated using the following equation:


(1) $$CI=SE*Z\left(0.95\right)$$

where SE = the standard error and *Z*(0.95) = the z-score corresponding to a confidence level of 0.95. Mann–Whitney U calculations were performed using GraphPad v8.1.2 (Prism; La Jolla, CA). *p*-values <0.05 were considered significant. Comparisons of metal levels across different regions in only control or only DLB brains were also carried out using Kruskal–Wallis tests in GraphPad v.8.1.2.

### Sensitivity analyses

In order to assess whether the interpretation of the data obtained in the current study was appropriate and robust, a sensitivity analysis was performed for every significant (*p* < 0.05) case–control difference in metal levels. For both individual runs and the mean values of all three replicate runs taken together, the risk ratio (RR), *E*-value, and effect size were determined. An explanation of these is given below.

The risk ratio is used to compare the risk of a ‘health event’ between different groups (in our case, to compare DLB cases and controls); it is determined by the following equation:


(2) $$RR=\left(\frac{a}{b}\right)/\left(\frac{c}{d}\right)$$

where *a* = the number of case values >95% upper CI limit of the controls (or < 95% lower CI limit where significant *decreases* were observed in cases), *b* = number of cases, *c* = number of control values >95% upper CI limit of the controls (or < 95% lower CI limit where significant *decreases* were observed in cases), and *d* = number of controls. Risk ratios of >3 were considered to be robust. In the case of null values in the calculation of risk ratios, the null values were assigned a value of 0.5.

*E*-values were calculated for risk ratios as well as for the upper and low confidence limits of the risk ratios. The *E*-value defines the minimum strength of association, on the risk ratio scale, that a potential confounder would have to have with both a treatment (e.g., metal/Cu levels) and an outcome (e.g., an increased risk of DLB) to explain away an observed treatment–outcome association, while taking into account measured covariates (here including age, sex, and PMD). The higher the *E*-value, the stronger the confounding required to nullify the treatment–outcome association. The *E*-value was calculated using Eq. (3):


(3) $$E\;\mathrm{value}=RR+\mathrm{sqrt}\;\left(RR\;\times \;\left(RR-1\right)\right)$$

In the calculation of *E*-values for RR < 1, the inverse of the risk ratio was first taken. *E*-values were also calculated for the confidence intervals of the risk ratios; if the range of the confidence intervals crossed 1.0, then the *E*-value was determined to be 1.0; otherwise, *E*-values were calculated according to Eq. 3, substituting RR for the CI closest to 1.0.

The effect size describes the *strength* of the relationship observed between variables, rather than indicating whether differences are present due to chance or otherwise. Determination of effect sizes can indicate where significantly altered (*p* < 0.05) variables have a negligible influence on an outcome, or conversely where variables found to be non-significant (*p* < 0.05) in traditional statistical testing have a large contribution towards an outcome; the latter may occur where statistical power is low due to small sample sizes. The effect size was here determined using Glass’ Delta:


(4) $$\mathrm{Glas}s^{\prime }\Delta =\left(M_{1}-M_{2}\right)/\mathrm{\sigma control}$$

where M_1_ = mean case value, M_2_ = mean control value, and σcontrol = standard deviation of the control group; M_1_ and M_2_ were reversed in case of significant decreases. Glass’ delta was used as the group sample sizes were equal, but their standard deviations were unequal. Effect size values 0.2–0.5 were considered small, values between 0.50 and 0.80 were considered of medium size, values between 0.80 and 1.30 were considered large, and effect sizes >1.30 were considered very large.

### PCA and PLS-DA

To determine whether the findings of the metallomic analysis were able to separate DLB cases from controls, principal component analysis (PCA) and principal least squares-discriminant analysis (PLS-DA) were carried out for each investigated brain region. PCA and PLS-DA analyses were carried out in MetaboAnalyst with log transformation and auto-scaling (mean-centring and division of the standard deviation of each variable). Metals with variable importance in projection (VIP) scores of >1.5 in the PLS-DA model were considered to contribute to case–control separation in that region.

### MANCOVA analysis

The potential effects of confounding factors such as tau Braak stage, age, sex, and PMD were investigated using two-way multivariate analysis of covariance (MANCOVA) tests in each region of the brain using SPSS (IBM SPSS Statistics 29.0; Warwick, UK). The test set-ups were as follows: design = intercept + age + PMD + Braak + Sex; dependent variables = Na, Mg, K, Ca, Mn, Fe, Cu, Zn, and Se; fixed factors = sex; covariates = age, PMD, and Braak. Wilks’ Lambda, *f* values, and *p*-values are reported; *p* < 0.05 was considered statistically significant.

### Comparisons with AD and PDD

The results of the DLB metallomic analysis were compared with those obtained for AD and PDD in previous studies (Xu et al., 2017; Scholefield et al., 2021). This was first done simply by comparing significant case–control findings from each disease study. Following this, PCA and PLS-DA were carried out to determine if metallomic data from regions investigated in both DLB and/or AD/PDD could be used to separate the different conditions. In the case of AD vs. DLB, six regions could be compared between the two: the MCX, CG, PVC, CB, HP, and MTG. In the case of AD vs. PDD, nine regions could be compared: the MCX, CG, PVC, CB, MED, PONS, HP, SN, and MTG. In the case of a comparison of DLB, AD, and PDD, six regions were investigated in all three diseases: the MCX, CG, PVC, CB, HP, and MTG.

Where separation was achieved, PLS-DA VIP scores were used to identify regions that contributed most to separation; using this information, regions were removed from the PCA until the fewest number of regions required for separation was identified.

## Data availability statement

The original contributions presented in the study are included in the article/Supplementary material, further inquiries can be directed to the corresponding author.

## Ethics statement

The studies involving humans were approved by Manchester REC, University of Manchester. The studies were conducted in accordance with the local legislation and institutional requirements. The human samples used in this study were acquired from the NIH NeuroBioBank brain tissue resource. Written informed consent for participation was not required from the participants or the participants’ legal guardians/next of kin in accordance with the national legislation and institutional requirements.

## Author contributions

MS: Conceptualization, Data curation, Formal analysis, Investigation, Methodology, Project administration, Visualization, Writing – original draft, Writing – review & editing. SC: Methodology, Validation, Writing – review & editing. JX: Investigation, Resources, Writing – review & editing. GC: Conceptualization, Funding acquisition, Supervision, Writing – review & editing.

## Supplementary material

Supplementary Material A Cohort characteristics, region summaries, additional PCA plots, and full MANCOVA results.

Supplementary Material B Raw data, mean averages, standard deviations, confidence intervals, statistical tests, and blanks.

## Glossary

AD: Alzheimer’s disease
ATP: Adenosine triphosphate
Ca: Calcium
CB: Cerebellum
CG: Cingulate gyrus
CI: Confidence interval
Cu: Copper
DLB: Dementia with Lewy bodies
fCX: Frontal cortex
FDG: Fluorodeoxyglucose
Fe: Iron
GABA: Gamma-aminobutyric acid
GPX: Glutathione peroxidase
HP: Hippocampus
ICP-MS: Inductively coupled plasma mass spectrometry
K: Potassium
LBD: Lewy body disease
MCX: Motor cortex
Mg: Magnesium
Mn: Manganese
MTG: Middle temporal gyrus
MTL: Middle temporal lobe
Na: Sodium
nCX: Neocortex
NMS: Neuroleptic malignant syndrome
PCA: Principal component analysis
PD: Parkinson’s disease
PDD: Parkinson’s disease dementia
PET: Positron emission tomography
PLS-DA: Partial least squares discriminant analysis
PMD: Post-mortem delay
PUT: Putamen
PVC: Primary visual cortex
RR: Risk ratio
SCX: Sensory cortex
SD: Standard deviation
Se: Selenium
SN: Substantia nigra
VIP: Variable importance point
Zn: Zinc

## References

[ref1] Akatsu H. Hori A. Yamamoto T. Yoshida M. Mimuro M. Hashizume Y.. (2012). Transition metal abnormalities in progressive dementias. Biometals 25, 337–350. doi: 10.1007/s10534-011-9504-8 22080191

[ref2] An Y. Li S. Huang X. Chen X. Shan H. Zhang M. (2022). The role of copper homeostasis in brain disease. Int. J. Mol. Sci. 23:13850. doi: 10.3390/ijms232213850, PMID: 36430330 PMC9698384

[ref3] Andersen K. B. Hansen A. K. Schacht A. C. Horsager J. Gottrup H. Klit H.. (2023). Synaptic density and glucose consumption in patients with Lewy body diseases: An [(11) C]Ucb-J and [(18) F]Fdg pet study. Mov. Disord. 38, 796–805. doi: 10.1002/mds.29375, PMID: 36905188

[ref4] Anderson J. P. Walker D. E. Goldstein J. M. De Laat R. Banducci K. Caccavello R. J.. (2006). Phosphorylation of Ser-129 is the dominant pathological modification of alpha-synuclein in familial and sporadic Lewy body disease. J. Biol. Chem. 281, 29739–29752. doi: 10.1074/jbc.M600933200, PMID: 16847063

[ref5] Ayton S. Lei P. Duce J. A. Wong B. X. Sedjahtera A. Adlard P. A.. (2013). Ceruloplasmin dysfunction and therapeutic potential for Parkinson disease. Ann. Neurol. 73, 554–559. doi: 10.1002/ana.23817, PMID: 23424051

[ref6] Barber R. Gholkar A. Scheltens P. Ballard C. Mckeith I. G. O'brien J. T. (1999). Medial temporal lobe atrophy on Mri in dementia with Lewy bodies. Neurology 52, 1153–1158. doi: 10.1212/WNL.52.6.1153 10214736

[ref7] Bayram E. Holden S. K. Fullard M. Armstrong M. J. Lewy Body Dementia Association Community Engagement Working, G (2023). Race and ethnicity in Lewy body dementia: a narrative review. J. Alzheimers Dis. 94, 861–878. doi: 10.3233/JAD-230207, PMID: 37355902 PMC10448838

[ref8] Braak H. Alafuzoff I. Arzberger T. Kretzschmar H. Del Tredici K. (2006). Staging of Alzheimer disease-associated neurofibrillary pathology using paraffin sections and immunocytochemistry. Acta Neuropathol. 112, 389–404. doi: 10.1007/s00401-006-0127-z, PMID: 16906426 PMC3906709

[ref9] Brenneisen P. Steinbrenner H. Sies H. (2005). Selenium, oxidative stress, and health aspects. Mol. Asp. Med. 26, 256–267. doi: 10.1016/j.mam.2005.07.004 16105679

[ref10] Burton E. J. Barber R. Mukaetova-Ladinska E. B. Robson J. Perry R. H. Jaros E.. (2009). Medial temporal lobe atrophy on Mri differentiates Alzheimer's disease from dementia with Lewy bodies and vascular cognitive impairment: a prospective study with pathological verification of diagnosis. Brain 132, 195–203. doi: 10.1093/brain/awn298, PMID: 19022858

[ref11] Corrigan F. M. Reynolds G. P. Ward N. I. (1993). Hippocampal tin, aluminum and zinc in Alzheimer's disease. Biometals 6, 149–154., PMID: 8400761 10.1007/BF00205853

[ref12] Costa-Mallen P. Gatenby C. Friend S. Maravilla K. R. Hu S. C. Cain K. C.. (2017). Brain iron concentrations in regions of interest and relation with serum iron levels in Parkinson disease. J. Neurol. Sci. 378, 38–44. doi: 10.1016/j.jns.2017.04.035, PMID: 28566175 PMC5609675

[ref13] Dalfo E. Ferrer I. (2008). Early alpha-synuclein lipoxidation in neocortex in Lewy body diseases. Neurobiol. Aging 29, 408–417. doi: 10.1016/j.neurobiolaging.2006.10.022, PMID: 17166629

[ref14] Dalfo E. Portero-Otin M. Ayala V. Martinez A. Pamplona R. Ferrer I. (2005). Evidence of oxidative stress in the neocortex in incidental Lewy body disease. J. Neuropathol. Exp. Neurol. 64, 816–830. doi: 10.1097/01.jnen.0000179050.54522.5a, PMID: 16141792

[ref15] Davies K. M. Bohic S. Carmona A. Ortega R. Cottam V. Hare D. J.. (2014). Copper pathology in vulnerable brain regions in Parkinson's disease. Neurobiol. Aging 35, 858–866. doi: 10.1016/j.neurobiolaging.2013.09.034, PMID: 24176624

[ref16] De Lores Arnaiz G. R. Ordieres M. G. (2014). Brain Na(+), K(+)-Atpase activity in aging and disease. Int. J. Biomed. Sci. 10, 85–102. doi: 10.59566/IJBS.2014.10085, PMID: 25018677 PMC4092085

[ref17] Deibel M. A. Ehmann W. D. Markesbery W. R. (1996). Copper, iron, and zinc imbalances in severely degenerated brain regions in Alzheimer's disease: possible relation to oxidative stress. J. Neurol. Sci. 143, 137–142. doi: 10.1016/S0022-510X(96)00203-1, PMID: 8981312

[ref18] Dexter D. T. Carayon A. Javoy-Agid F. Agid Y. Wells F. R. Daniel S. E.. (1991). Alterations in the levels of iron, ferritin and other trace metals in Parkinson's disease and other neurodegenerative diseases affecting the basal ganglia. Brain 114, 1953–1975. doi: 10.1093/brain/114.4.1953, PMID: 1832073

[ref19] Dexter D. T. Sian J. Rose S. Hindmarsh J. G. Mann V. M. Cooper J. M.. (1994). Indices of oxidative stress and mitochondrial function in individuals with incidental Lewy body disease. Ann. Neurol. 35, 38–44. doi: 10.1002/ana.410350107, PMID: 8285590

[ref20] Fujishiro H. Iseki E. Kasanuki K. Murayama N. Ota K. Suzuki M.. (2012). Glucose hypometabolism in primary visual cortex is commonly associated with clinical features of dementia with Lewy bodies regardless of cognitive conditions. Int. J. Geriatr. Psychiatry 27, 1138–1146. doi: 10.1002/gps.2836, PMID: 22250011

[ref21] Galaris D. Barbouti A. Pantopoulos K. (2019). Iron homeostasis and oxidative stress: An intimate relationship. Biochim. Biophys. Acta, Mol. Cell Res. 1866:118535. doi: 10.1016/j.bbamcr.2019.118535, PMID: 31446062

[ref22] Genoud S. Roberts B. R. Gunn A. P. Halliday G. M. Lewis S. J. G. Ball H. J.. (2017). Subcellular compartmentalisation of copper, iron, manganese, and zinc in the Parkinson's disease brain. Metallomics 9, 1447–1455. doi: 10.1039/C7MT00244K, PMID: 28944802 PMC5647261

[ref23] Gomez-Tortosa E. Sanders J. L. Newell K. Hyman B. T. (2001). Cortical neurons expressing calcium binding proteins are spared in dementia with Lewy bodies. Acta Neuropathol. 101, 36–42. doi: 10.1007/s004010000270, PMID: 11194939

[ref24] Graham S. F. Nasarauddin M. B. Carey M. Mcguinness B. Holscher C. Kehoe P. G.. (2015). Quantitative measurement of [Na+] and [K+] in postmortem human brain tissue indicates disturbances in subjects with Alzheimer's disease and dementia with Lewy bodies. J. Alzheimers Dis. 44, 851–857. doi: 10.3233/JAD-141869, PMID: 25362038

[ref25] Graham S. F. Nasaruddin M. B. Carey M. Holscher C. Mcguinness B. Kehoe P. G.. (2014). Age-associated changes of brain copper, iron, and zinc in Alzheimer's disease and dementia with Lewy bodies. J. Alzheimers Dis. 42, 1407–1413. doi: 10.3233/JAD-140684, PMID: 25024342

[ref26] Harris J. J. Jolivet R. Attwell D. (2012). Synaptic energy use and supply. Neuron 75, 762–777. doi: 10.1016/j.neuron.2012.08.019 22958818

[ref27] Hershey L. A. Coleman-Jackson R. (2019). Pharmacological Management of Dementia with Lewy bodies. Drugs Aging 36, 309–319. doi: 10.1007/s40266-018-00636-7, PMID: 30680679 PMC6435621

[ref28] Higuchi M. Tashiro M. Arai H. Okamura N. Hara S. Higuchi S.. (2000). Glucose hypometabolism and neuropathological correlates in brains of dementia with Lewy bodies. Exp. Neurol. 162, 247–256. doi: 10.1006/exnr.2000.7342, PMID: 10739631

[ref29] Imabayashi E. Yokoyama K. Tsukamoto T. Sone D. Sumida K. Kimura Y.. (2016). The cingulate island sign within early Alzheimer's disease-specific hypoperfusion volumes of interest is useful for differentiating Alzheimer's disease from dementia with Lewy bodies. EJNMMI Res. 6:67. doi: 10.1186/s13550-016-0224-5, PMID: 27620458 PMC5020033

[ref30] Irwin D. J. Grossman M. Weintraub D. Hurtig H. I. Duda J. E. Xie S. X.. (2017). Neuropathological and genetic correlates of survival and dementia onset in synucleinopathies: a retrospective analysis. Lancet Neurol. 16, 55–65. doi: 10.1016/S1474-4422(16)30291-5, PMID: 27979356 PMC5181646

[ref31] Ishii K. Imamura T. Sasaki M. Yamaji S. Sakamoto S. Kitagaki H.. (1998). Regional cerebral glucose metabolism in dementia with Lewy bodies and Alzheimer's disease. Neurology 51, 125–130. doi: 10.1212/WNL.51.1.125 9674790

[ref32] Jellinger K. A. Korczyn A. D. (2018). Are dementia with Lewy bodies and Parkinson's disease dementia the same disease? BMC Med. 16:34. doi: 10.1186/s12916-018-1016-8, PMID: 29510692 PMC5840831

[ref33] Kawamoto E. M. Vivar C. Camandola S. (2012). Physiology and pathology of calcium signaling in the brain. Front. Pharmacol. 3:61. doi: 10.3389/fphar.2012.00061 22518105 PMC3325487

[ref34] Khundakar A. A. Hanson P. S. Erskine D. Lax N. Z. Roscamp J. Karyka E.. (2016). Analysis of primary visual cortex in dementia with Lewy bodies indicates Gabaergic involvement associated with recurrent complex visual hallucinations. Acta Neuropathol. Commun. 4:66. doi: 10.1186/s40478-016-0334-3, PMID: 27357212 PMC4928325

[ref35] Koukouraki P. Doxakis E. (2016). Constitutive translation of human alpha-synuclein is mediated by the 5′-untranslated region. Open Biol. 6:160022. doi: 10.1098/rsob.160022, PMID: 27248657 PMC4852460

[ref36] Kurasz A. M. De Wit L. Smith G. E. Armstrong M. J. (2022). Neuropathological and clinical correlates of Lewy body disease survival by race and ethnicity in the National Alzheimer's coordinating center. J. Alzheimers Dis. 89, 1339–1349. doi: 10.3233/JAD-220297, PMID: 36031892 PMC9588566

[ref37] Kurasz A. M. Smith G. E. Mcfarland M. G. Armstrong M. J. (2020). Ethnoracial differences in Lewy body diseases with cognitive impairment. J. Alzheimers Dis. 77, 165–174. doi: 10.3233/JAD-200395, PMID: 32804137 PMC7553012

[ref38] Levin J. Hogen T. Hillmer A. S. Bader B. Schmidt F. Kamp F.. (2011). Generation of ferric iron links oxidative stress to alpha-synuclein oligomer formation. J. Parkinsons Dis. 1, 205–216. doi: 10.3233/JPD-2011-11040, PMID: 23934922

[ref39] Li L. Yang X. (2018). The essential element manganese, oxidative stress, and metabolic diseases: links and interactions. Oxidative Med. Cell. Longev. 2018:7580707. doi: 10.1155/2018/7580707 PMC5907490 29849912

[ref40] Lippa C. F. Johnson R. Smith T. W. (1998). The medial temporal lobe in dementia with Lewy bodies: a comparative study with Alzheimer's disease. Ann. Neurol. 43, 102–106. doi: 10.1002/ana.410430117, PMID: 9450774

[ref41] Loeffler D. A. Lewitt P. A. Juneau P. L. Sima A. A. Nguyen H. U. Demaggio A. J.. (1996). Increased regional brain concentrations of ceruloplasmin in neurodegenerative disorders. Brain Res. 738, 265–274. doi: 10.1016/S0006-8993(96)00782-2, PMID: 8955522

[ref42] Lu Y. Prudent M. Fauvet B. Lashuel H. A. Girault H. H. (2011). Phosphorylation of alpha-Synuclein at Y125 and S129 alters its metal binding properties: implications for understanding the role of alpha-Synuclein in the pathogenesis of Parkinson's disease and related disorders. ACS Chem. Neurosci. 2, 667–675. doi: 10.1021/cn200074d, PMID: 22860160 PMC3369716

[ref43] Lyras L. Perry R. H. Perry E. K. Ince P. G. Jenner A. Jenner P.. (1998). Oxidative damage to proteins, lipids, and Dna in cortical brain regions from patients with dementia with Lewy bodies. J. Neurochem. 71, 302–312. doi: 10.1046/j.1471-4159.1998.71010302.x, PMID: 9648879

[ref44] Magaki S. Raghavan R. Mueller C. Oberg K. C. Vinters H. V. Kirsch W. M. (2007). Iron, copper, and iron regulatory protein 2 in Alzheimer's disease and related dementias. Neurosci. Lett. 418, 72–76. doi: 10.1016/j.neulet.2007.02.077, PMID: 17408857 PMC1955223

[ref45] Mckeith I. G. Boeve B. F. Dickson D. W. Halliday G. Taylor J. P. Weintraub D.. (2017). Diagnosis and management of dementia with Lewy bodies: fourth consensus report of the Dlb consortium. Neurology 89, 88–100. doi: 10.1212/WNL.0000000000004058, PMID: 28592453 PMC5496518

[ref46] Navarro A. Boveris A. Bandez M. J. Sanchez-Pino M. J. Gomez C. Muntane G.. (2009). Human brain cortex: mitochondrial oxidative damage and adaptive response in Parkinson disease and in dementia with Lewy bodies. Free Radic. Biol. Med. 46, 1574–1580. doi: 10.1016/j.freeradbiomed.2009.03.007, PMID: 19298851

[ref47] Nielsen M. S. Vorum H. Lindersson E. Jensen P. H. (2001). Ca2+ binding to alpha-synuclein regulates ligand binding and oligomerization. J. Biol. Chem. 276, 22680–22684. doi: 10.1074/jbc.M101181200, PMID: 11312271

[ref48] Nunomura A. Chiba S. Kosaka K. Takeda A. Castellani R. J. Smith M. A.. (2002). Neuronal Rna oxidation is a prominent feature of dementia with Lewy bodies. Neuroreport 13, 2035–2039. doi: 10.1097/00001756-200211150-00009, PMID: 12438921

[ref49] Ortega R. Carmona A. Roudeau S. Perrin L. Ducic T. Carboni E.. (2016). Alpha-Synuclein over-expression induces increased Iron accumulation and redistribution in Iron-exposed neurons. Mol. Neurobiol. 53, 1925–1934. doi: 10.1007/s12035-015-9146-x, PMID: 25833099

[ref50] Patterson L. Firbank M. J. Colloby S. J. Attems J. Thomas A. J. Morris C. M. (2019). Neuropathological changes in dementia with Lewy bodies and the Cingulate Island sign. J. Neuropathol. Exp. Neurol. 78, 717–724. doi: 10.1093/jnen/nlz047, PMID: 31271438 PMC6640897

[ref51] Peng Y. Wang C. Xu H. H. Liu Y. N. Zhou F. (2010). Binding of alpha-synuclein with Fe(iii) and with Fe(ii) and biological implications of the resultant complexes. J. Inorg. Biochem. 104, 365–370. doi: 10.1016/j.jinorgbio.2009.11.005, PMID: 20005574 PMC2824027

[ref52] Power J. H. Blumbergs P. C. (2009). Cellular glutathione peroxidase in human brain: cellular distribution, and its potential role in the degradation of Lewy bodies in Parkinson's disease and dementia with Lewy bodies. Acta Neuropathol. 117, 63–73. doi: 10.1007/s00401-008-0438-3, PMID: 18853169

[ref53] Power J. H. Shannon J. M. Blumbergs P. C. Gai W. P. (2002). Nonselenium glutathione peroxidase in human brain: elevated levels in Parkinson's disease and dementia with lewy bodies. Am. J. Pathol. 161, 885–894. doi: 10.1016/S0002-9440(10)64249-6, PMID: 12213717 PMC1867235

[ref54] Rajkumar A. P. Bidkhori G. Shoaie S. Clarke E. Morrin H. Hye A.. (2020). Postmortem cortical transcriptomics of Lewy body dementia reveal mitochondrial dysfunction and lack of Neuroinflammation. Am. J. Geriatr. Psychiatry 28, 75–86. doi: 10.1016/j.jagp.2019.06.007, PMID: 31327631

[ref55] Raven E. P. Lu P. H. Tishler T. A. Heydari P. Bartzokis G. (2013). Increased iron levels and decreased tissue integrity in hippocampus of Alzheimer's disease detected in vivo with magnetic resonance imaging. J. Alzheimers Dis. 37, 127–136. doi: 10.3233/JAD-130209, PMID: 23792695

[ref56] Religa D. Strozyk D. Cherny R. A. Volitakis I. Haroutunian V. Winblad B.. (2006). Elevated cortical zinc in Alzheimer disease. Neurology 67, 69–75. doi: 10.1212/01.wnl.0000223644.08653.b5 16832080

[ref57] Ruiz L. M. Libedinsky A. Elorza A. A. (2021). Role of copper on mitochondrial function and metabolism. Front. Mol. Biosci. 8:711227. doi: 10.3389/fmolb.2021.711227, PMID: 34504870 PMC8421569

[ref58] Santulli G. Marks A. R. (2015). Essential roles of intracellular calcium release channels in muscle, brain, metabolism, and aging. Curr. Mol. Pharmacol. 8, 206–222. doi: 10.2174/1874467208666150507105105, PMID: 25966694

[ref59] Scholefield M. Church S. J. Xu J. Kassab S. Gardiner N. J. Roncaroli F.. (2020). Evidence that levels of nine essential metals in post-mortem human-Alzheimer's-brain and ex vivo rat-brain tissues are unaffected by differences in post-mortem delay, age, disease staging, and brain bank location. Metallomics 12, 952–962. doi: 10.1039/d0mt00048e, PMID: 32373908

[ref60] Scholefield M. Church S. J. Xu J. Patassini S. Roncaroli F. Hooper N. M.. (2021). Widespread decreases in cerebral copper are common to Parkinson's disease dementia and Alzheimer's disease dementia. Front. Aging Neurosci. 13:641222. doi: 10.3389/fnagi.2021.641222, PMID: 33746735 PMC7966713

[ref61] Szabo S. T. Harry G. J. Hayden K. M. Szabo D. T. Birnbaum L. (2016). Comparison of metal levels between postmortem brain and ventricular fluid in Alzheimer's disease and nondemented elderly controls. Toxicol. Sci. 150, 292–300. doi: 10.1093/toxsci/kfv325, PMID: 26721301 PMC4881830

[ref62] Twohig D. Nielsen H. M. (2019). Alpha-synuclein in the pathophysiology of Alzheimer's disease. Mol. Neurodegener. 14:23. doi: 10.1186/s13024-019-0320-x, PMID: 31186026 PMC6558879

[ref63] Vitvitsky V. M. Garg S. K. Keep R. F. Albin R. L. Banerjee R. (2012). Na+ and K+ ion imbalances in Alzheimer's disease. Biochim. Biophys. Acta 1822, 1671–1681. doi: 10.1016/j.bbadis.2012.07.004, PMID: 22820549 PMC3444663

[ref64] Winterbourn C. C. (1995). Toxicity of iron and hydrogen peroxide: the Fenton reaction. Toxicol. Lett. 82-83, 969–974. doi: 10.1016/0378-4274(95)03532-X 8597169

[ref65] Xu J. Church S. J. Patassini S. Begley P. Waldvogel H. J. Curtis M. A.. (2017). Evidence for widespread, severe brain copper deficiency in Alzheimer's dementia. Metallomics 9, 1106–1119. doi: 10.1039/C7MT00074J, PMID: 28654115

