# Supplementary material for: Metallomic analysis of brain tissues distinguishes between cases of dementia with Lewy bodies, Alzheimer’s disease, and Parkinson’s disease dementia
Source: Front Neurosci. 2024 Jun 26;18:1412356. doi: 10.3389/fnins.2024.1412356 (PMC11233441; doi:10.3389/fnins.2024.1412356)
Supplement: Supplementary file 1 [file Data_Sheet_1.zip › Supplementary Material A.DOCX]

Supplementary Material A

# **Suppl. Table 1. Cohort Characteristics**

| **ID** | **Source** | **Type** | **Age** | **Sex** | **PMD** | **Race** | **Clinical Brain Diagnosis** | **DLB Type** | **α-syn Braak stage** | **tau Braak stage** | **Comorbidities** |
| --- | --- | --- | --- | --- | --- | --- | --- | --- | --- | --- | --- |
| **C1** | Sepulveda | C | 79 | Female | 14 | White | No Dementia | N/A | 0 | 0 | Coronary Artery Disease, hypertension |
| **C2** | Sepulveda | C | 72 | Male | 12.2 | White | No Dementia | N/A | 0 | 1 | COPD, Pulmonary Emphysema |
| **C3** | Sepulveda | C | 65 | Female | 17.3 | White | No Dementia | N/A | 0 | 0 | None reported |
| **C4** | Harvard | C | 69 | Female | 21.3 | Unknown | No Dementia | N/A | 0 | 1 | Mild atherosclerosis and arteriosclerosis |
| **C5** | Harvard | C | 66 | Male | 20.9 | White | No Dementia | N/A | 0 | 1 | Argyrophilic grain disease (mild), mild arteriosclerosis |
| **C6** | Harvard | C | 74 | Female | 16.6 | Unknown | No Dementia | N/A | 0 | 2 | Reactive gliosis |
| **C7** | Harvard | C | 74 | Male | 14.3 | Unknown | No Dementia | N/A | 0 | 2 | CVD, atherosclerosis, arteriosclerosis |
| **C8** | Harvard | C | 73 | Male | 18.0 | White | No Dementia | N/A | 0 | 1 | Atherosclerosis, arteriosclerosis |
| **C9** | Harvard | C | 68 | Male | 19.2 | Unknown | No Dementia | N/A | 0 | 2 | SVD, atherosclerosis, arteriosclerosis, small infarct in pons, three microinfarcts in frontal lobe white matter |
| **C10** | Harvard | C | 66 | Male | 19.4 | White | No Dementia | N/A | 0 | 1 | Arteriosclerosis |
| **C11** | Harvard | C | 70 | Female | 17.2 | White | No Dementia | N/A | 0 | 1 | Arteriosclerosis, mild autolysis |
| **C12** | Harvard | C | 76 | Female | 8.1 | Black/African-American | No Dementia | N/A | 0 | 1 | Remote probable tentorial notching of ventral uncus, arteriosclerosis, arterial intimal hyperplasia |
| **C13** | Harvard | C | 71 | Female | 14.1 | Unknown | No Dementia | N/A | 0 | 1 | None reported |
| **C14** | Harvard | C | 85 | Male | 20.8 | White | No Dementia | N/A | 0 | 2 | Atherosclerosis, arteriosclerosis, remote microhaemorrhage in substantia nigra |
| **C15** | Harvard | C | 77 | Male | 14.6 | Unknown | No Dementia | N/A | 0 | 2 | Atherosclerosis, arteriosclerosis |
| **C16** | Harvard | C | 85 | Male | 29.1 | Unknown | No Dementia | N/A | 0 | 1 | Atherosclerosis, arteriosclerosis, mild autolysis |
| **C17** | Harvard | C | 78 | Female | 22.7 | Unknown | No Dementia | N/A | 0 | 1 | CVD, atherosclerosis, arteriosclerosis, remote small infarcts |
| **C18** | Harvard | C | 66 | Male | 22.1 | White | No Dementia | N/A | 0 | 1 | Mild arteriosclerosis, mild to moderate autolysis |
| **C19** | Harvard | C | 84 | Male | 28.8 | White | No Dementia | N/A | 0 | 1 | Amyloid angiopathy, atherosclerosis, arteriosclerosis, mild Purkinje cell loss, mild autolysis |
| **C20** | Harvard | C | 74 | Male | 18.6 | Unknown | No Dementia | N/A | 0 | 0 | None reported |
| **C21** | Harvard | C | 65 | Male | 15.8 | Unknown | No Dementia | N/A | 0 | *0/1** | None reported |
| **C22** | Harvard | C | 68 | Male | 16.1 | Unknown | No Dementia | N/A | 0 | *0/1** | None reported |
| **C23** | Harvard | C | 83 | Male | 13.0 | Unknown | No Dementia | N/A | 0 | *0/1** | None reported |
| **DLB1** | Sepulveda | DLB | 78 | Male | 10 | White | DLB | *Limbic** | Unknown | *2** | None reported |
| **DLB2** | Sepulveda | DLB | 75 | Male | 11.9 | White | DLB/AD | *Limbic** | Unknown | 4/5 | Carcinoma in situ of bladder, hypertension |
| **DLB3** | Sepulveda | DLB | 68 | Male | 11.9 | White | DLB/AD | Diffuse  /cortical | Unknown | 2/3 | Hallucinations, paranoid personality disorder, atherosclerotic heart disease of native coronary artery, hypertension, type II diabetes mellitus |
| **DLB4** | Sepulveda | DLB | 80 | Female | 12.8 | White | DLB/AD | Cortical | Unknown | 2/3 | Osteoporosis, pneumonia, anxiety disorder, major depressive disorder |
| **DLB5** | Sepulveda | DLB | 71 | Male | 20.3 | White | DLB | *Limbic** | Unknown | 0 | Alcohol abuse, major depressive disorder, hypertension, sleep disorder, type II diabetes mellitus |
| **DLB6** | Sepulveda | DLB | 66 | Male | 17.3 | White | DLB | Limbic | Unknown | 0 | Chronic kidney disease, diabetes mellitus, anxiety disorder, hallucinations, major depressive disorder |
| **DLB7** | Sepulveda | DLB | 68 | Female | 8 | White | DLB/AD | Diffuse | Unknown | 5 | None reported |
| **DLB8** | Harvard | DLB | 74 | Male | 11.0 | White | DLB/AD | Cortical | Unknown | 5 | Amyloid angiopathy, SVD, arteriosclerosis |
| **DLB9** | Harvard | DLB | 71 | Female | 9.6 | White | DLB/AD | Cortical | Unknown | 4 | Mild atherosclerosis, arteriosclerosis, minute capillary telangiectasia |
| **DLB10** | Harvard | DLB | 73 | Male | 11.3 | White | DLB | Cortical | 5 | 2 | Atherosclerosis, arteriosclerosis, single minute focus of probable fat embolism (history of hip trauma) |
| **DLB11** | Harvard | DLB | 85 | Female | 8.6 | White | DLB | Limbic | Unknown | 5 | Amyloid angiopathy, arteriosclerosis |
| **DLB12** | Harvard | DLB | 76 | Female | 12.8 | White | DLB/AD | Limbic | Unknown | 3 | SVD, arteriosclerosis, atherosclerosis, multiple microinfarcts |
| **DLB13** | Harvard | DLB | 75 | Female | 22.6 | White | DLB | Diffuse | Unknown | 2/3 | Very mild amyloid angiopathy |
| **DLB14** | Harvard | DLB | 72 | Female | 18.8 | White | DLB/AD | Diffuse | 6 | 6 | TDP-43 proteinopathy, CVD, mild arteriosclerosis |
| **DLB15** | Harvard | DLB | 65 | Male | 19.2 | White | DLB | Limbic | 4 | 2 | Atherosclerosis, mild arteriosclerosis |
| **DLB16** | Harvard | DLB | 73 | Female | 10.4 | White | DLB | Cortical | Unknown | 4 | Atherosclerosis, arteriosclerosis |
| **DLB17** | Harvard | DLB | 77 | Male | 15.0 | White | DLB | Cortical | 5 | 4 | Moderate to severe amyloid angiopathy, SVD, atherosclerosis, arteriosclerosis, microinfarcts |
| **DLB18** | Harvard | DLB | 79 | Male | 20.6 | White | DLB/AD | *Limbic** | Unknown | 6 | CVD, remote infarcts, cerebral amyloid angiopathy, arteriolar sclerosis, moderate to severe atherosclerosis, COVID-19 (in remission), atherosclerotic heart disease of native coronary artery, chronic kidney disease, chronic obstructive pulmonary disease, heart failure, hypertension, type II diabetes mellitus, unspecified atrial fibrillation |
| **DLB19** | Harvard | DLB | 80 | Male | 20.0 | White | DLB | Cortical | Unknown | 3 | Mild chronic traumatic encephalopathy, small fibrous meningioma, mild remote subdural haemorrhage with subdural membrane, atherosclerosis, arteriosclerosis, benign prostatic hyperplasia with lower urinary tract symptoms |
| **DLB20** | Harvard | DLB | 76 | Male | 21.3 | White | DLB | Diffuse | 5 | 2 | CVD, arteriolar sclerosis, chronic mastoiditis (left ear), hyperlipidaemia |

**Putative staging based on medical reports supplied by brain bank.*

AD = Alzheimer’s disease; DLB = Dementia with Lewy bodies; N/A = Not applicable; PMD = Post-mortem delay

# **Suppl. Table 2. Cohort Region Summaries**

|  | **Age at Death (years)** | **Sex (% Male)** | **PMD (hours)** |
| --- | --- | --- | --- |
|  |  | **HP** |  |
| Controls (n = 14) | 73.6 (65-85) | 35.7 | 17.6 (12.2-28.8) |
| Cases (n = 15) | 73.1 (65-85) | 43.8 | 13.7 (8.0-23.6)* |
|  | **MED** | | |
| Controls (n = 15) | 72.3 (65-85) | 46.7 | 16.3 (8.1-21.3) |
| Cases (n = 15) | 73.1 (65-85) | 43.8 | 13.7 (8.0-23.6) |
|  | **MTG** | | |
| Controls (n = 15) | 72.8 (65-85) | 40.0 | 16.9 (12.2-21.3) |
| Cases (n = 15) | 73.1 (65-85) | 43.8 | 13.7 (8.0-23.6)* |
|  | **CG** | | |
| Controls (n = 15) | 73.0 (65-85) | 46.7 | 16.2 (8.1-21.3) |
| Cases (n = 15) | 73.1 (65-85) | 43.8 | 13.7 (8.0-23.6) |
|  | **PVC** | | |
| Controls (n = 16) | 73.0 (65-85) | 43.8 | 16.3 (8.1-21.3) |
| Cases (n = 15) | 73.1 (65-85) | 43.8 | 13.7 (8.0-23.6) |
|  | **PONS** | | |
| Controls (n = 14) | 71.6 (65-85) | 35.7 | 16.9 (8.1-21.3) |
| Cases (n = 8) | 73.9 (65-85) | 55.6 | 14.2 (8.6-23.6) |
|  | **MCX** | | |
| Controls (n = 16) | 73.0 (65-85) | 43.8 | 16.3 (8.1-21.3) |
| Cases (n = 15) | 73.1 (65-85) | 43.8 | 13.7 (8.6-23.6) |
|  | **PUT** | | |
| Controls (n = 13) | 71.8 (65-85) | 46.2 | 17.0 (8.1-21.3) |
| Cases (n = 8) | 73.1 (65-85) | 43.8 | 13.7 (8.6-23.6) |
|  | **SN** | | |
| Controls (n = 18) | 73.7 (65-85) | 38.9 | 18.2 (8.1-29.1) |
| Cases (n = 15) | 73.1 (65-85) | 43.8 | 13.7 (8.6-23.6)* |
|  |  | **CB** |  |
| Controls (n = 16) | 73.7 (66-85) | 38.5 | 18.8 (8.1-28.8) |
| Cases (n = 12) | 75.0 (65-85) | 38.5 | 15.7 (8.6-23.6) |

Values shown for age at death and post-mortem delay (PMD) are means (range). * p < 0.05 as determined by Welch’s t-test.

# **Suppl. Figure 1. DLB vs PDD PCA Plots**

a)


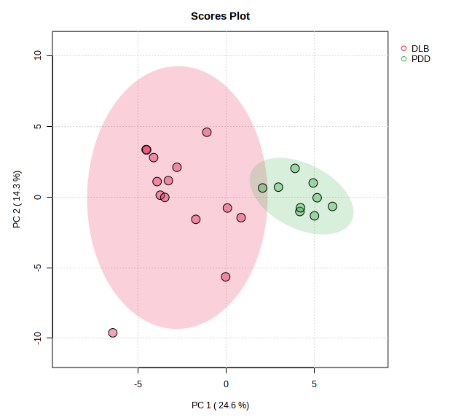

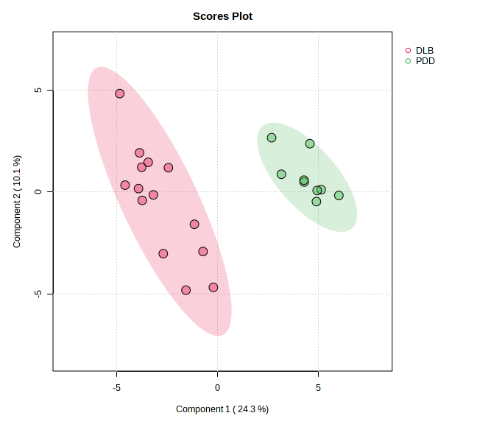

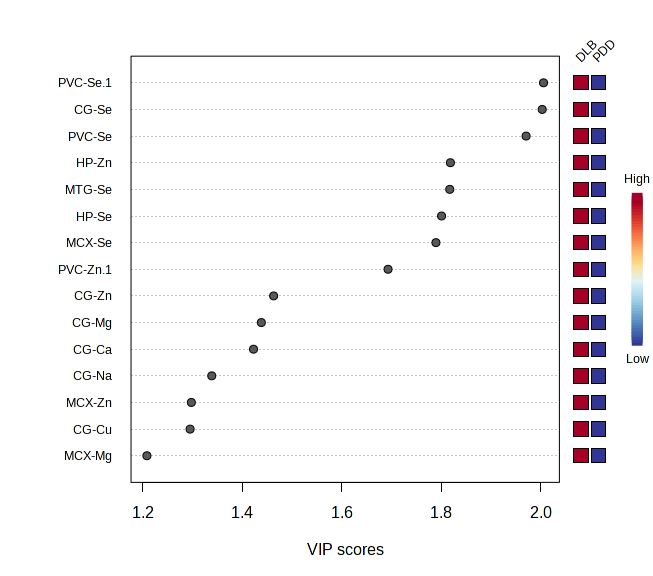


b)


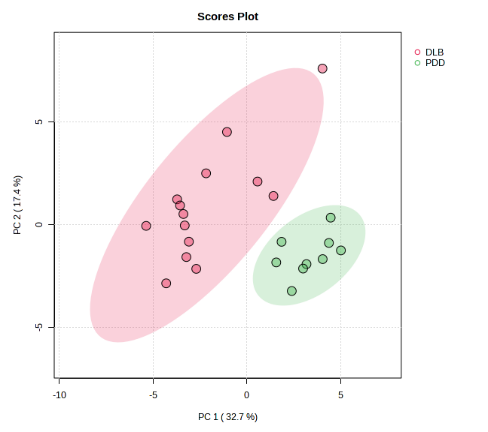

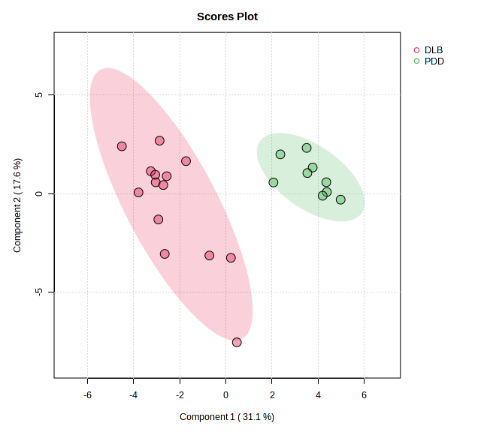

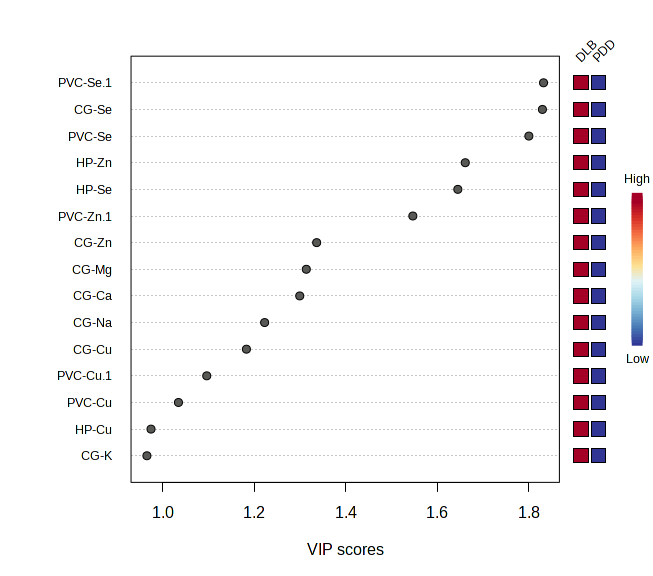


a) DLB vs PDD cases using data from CG, HP, MTG, MCX, MED, SN and PVC. b) DLB vs PDD cases using data from PVC, CG, and HP. Plots are ordered as follows: PCA plot, PLS-DA plot, VIP scores of PLS-DA plot. DLB cases are shown in red and PDD cases in green.

# **Suppl. Figure 2. DLB vs AD PCA Plots**


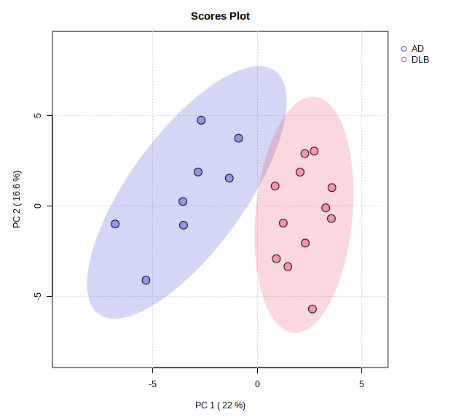

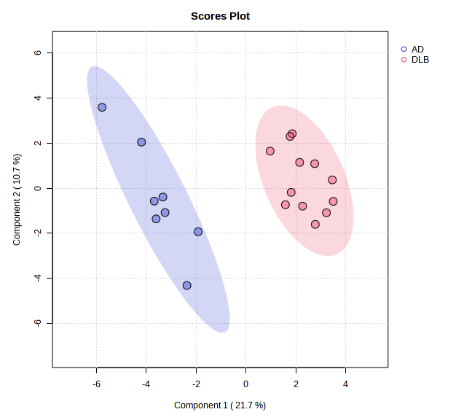

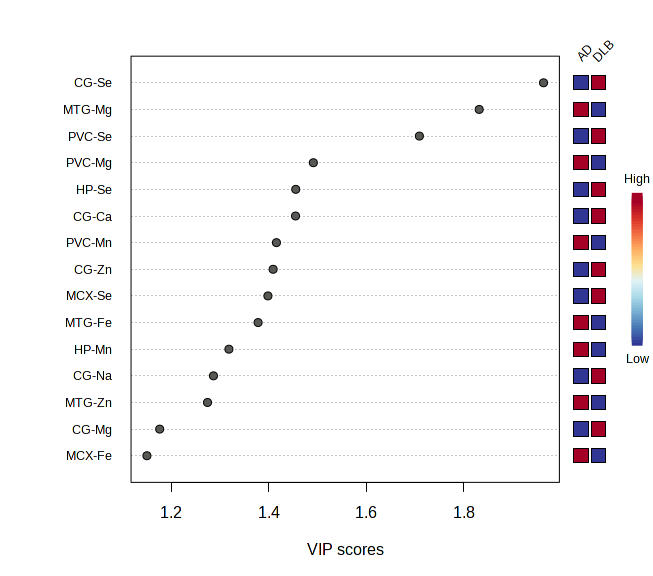


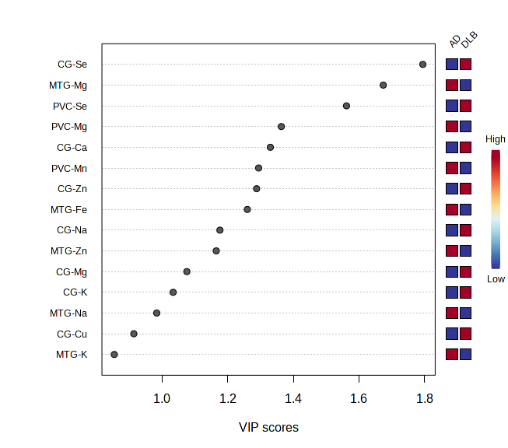


a) DLB vs AD using data from CG, HP, MTG, PVC, and MCX. b) VIP scores for DLB vs AD using data from CG, MTG, and PVC. c) DLB vs AD using data from CG and MTG. Plots in a) and c) are ordered as follows: PCA plot, PLS-DA plot, VIP scores of PLS-DA plot. DLB cases are shown in red and PDD cases in green.

# **Suppl. Figure 3. DLB vs PDD vs AD PCA Plots**


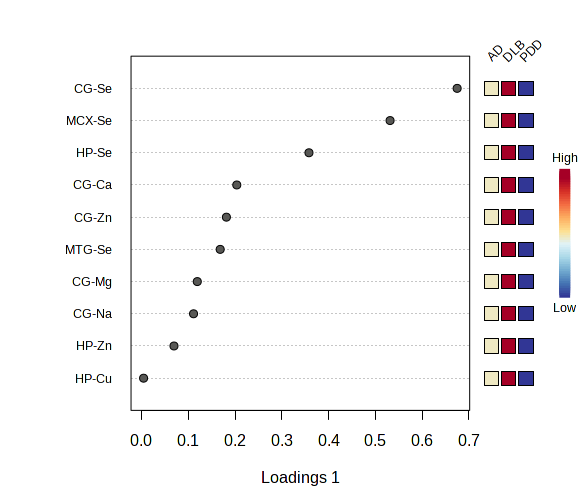


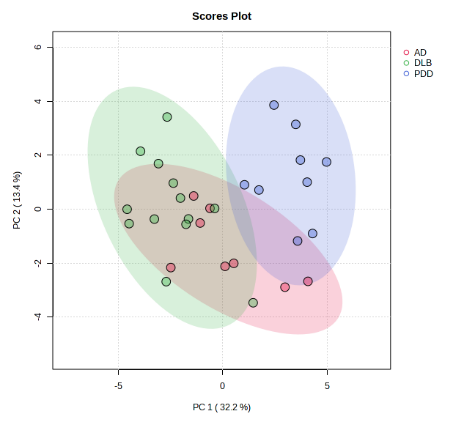

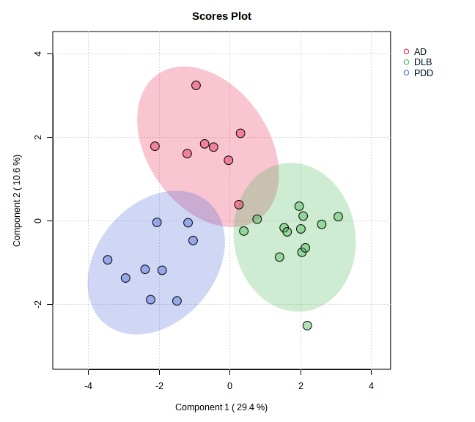

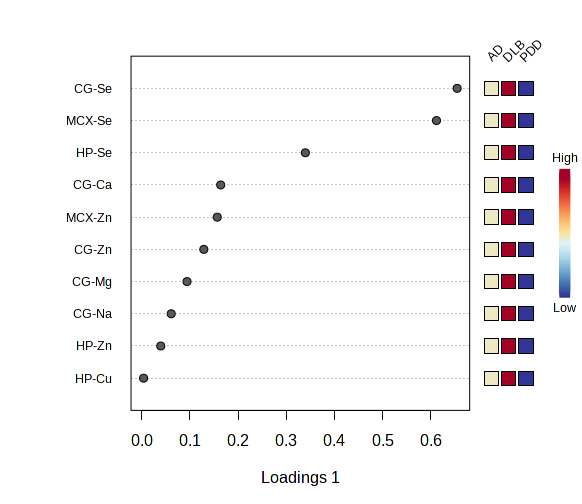


a) VIP scores for DLB vs PDD vs AD cases using data from CG, HP, MTG, and MCX. b) DLB vs PDD vs AD cases using data from MTG, MCX, and HP. Plots are ordered as follows: PCA plot, PLS-DA plot, VIP scores of PLS-DA plot. DLB cases are shown in red and PDD cases in green.

# **Suppl. Table 3. MANCOVA Results—Overall**

| Effect | Wilks’ Lambda (Λ) | F | P-Value |
| --- | --- | --- | --- |
| CB | | | |
| Intercept | 0.5 | 0.9 | 0.6 |
| Age | 0.5 | 1.1 | 0.5 |
| PMD | 0.5 | 1.2 | 0.4 |
| Braak | 0.1 | 6.2 | 0.006 |
| Sex | 0.6 | 0.8 | 0.6 |
| CG | | | |
| Intercept | 0.5 | 0.5 | 0.8 |
| Age | 0.5 | 0.6 | 0.8 |
| PMD | 0.4 | 0.9 | 0.6 |
| Braak | 0.08 | 6.7 | 0.03 |
| Sex | 0.2 | 2.4 | 0.2 |
| HP | | | |
| Intercept | 0.4 | 0.8 | 0.6 |
| Age | 0.2 | 1.7 | 0.3 |
| PMD | 0.3 | 1.2 | 0.5 |
| Braak | 0.1 | 2.9 | 0.2 |
| Sex | 0.2 | 2.5 | 0.2 |
| MCX | | | |
| Intercept | 0.5 | 0.7 | 0.7 |
| Age | 0.4 | 1.3 | 0.4 |
| PMD | 0.3 | 2.3 | 0.1 |
| Braak | 0.2 | 3.4 | 0.06 |
| Sex | 0.7 | 0.3 | 0.9 |
| MED | | | |
| Intercept | 0.05 | 5.9 | 0.09 |
| Age | 0.03 | 10.3 | 0.04 |
| PMD | 0.05 | 6.3 | 0.08 |
| Braak | 0.1 | 2.6 | 0.2 |
| Sex | 0.04 | 9.1 | 0.05 |
| MTG | | | |
| Intercept | 0.5 | 0.7 | 0.7 |
| Age | 0.3 | 1.4 | 0.3 |
| PMD | 0.3 | 1.7 | 0.3 |
| Braak | 0.2 | 3.6 | 0.07 |
| Sex | 0.4 | 1.1 | 0.5 |
| PONS | | | |
| Intercept | 0.5 | 0.7 | 0.7 |
| Age | 0.5 | 0.7 | 0.7 |
| PMD | 0.6 | 0.6 | 0.8 |
| Braak | 0.2 | 2.8 | 0.1 |
| Sex | 0.5 | 0.7 | 0.7 |
| PUT | | | |
| Intercept | 0.3 | 1.2 | 0.5 |
| Age | 0.3 | 1.2 | 0.5 |
| PMD | 0.7 | 0.2 | 1.0 |
| Braak | 0.1 | 1.7 | 0.2 |
| Sex | 0.6 | 0.3 | 0.9 |
| PVC | | | |
| Intercept | 0.2 | 2.8 | 0.1 |
| Age | 0.3 | 1.8 | 0.2 |
| PMD | 0.5 | 0.7 | 0.7 |
| Braak | 0.3 | 1.9 | 0.2 |
| Sex | 0.5 | 0.6 | 0.8 |
| SN | | | |
| Intercept | 0.3 | 2.6 | 0.08 |
| Age | 0.8 | 0.2 | 1.0 |
| PMD | 0.5 | 1.0 | 0.5 |
| Braak | 0.4 | 1.8 | 0.2 |
| Sex | 0.6 | 0.6 | 0.8 |

**Suppl. Table 4.** MANCOVA analysis of overall covariate effects in different brain regions. Design: Intercept + Age + tau Braak Stage + PMD + Sex. p < 0.05 considered significant.

# **Suppl. Table 4. MANCOVA Results—Significant Effects on Individual Metals**

| Effect | F | P-Value |
| --- | --- | --- |
| PONS | | |
| Tau Braak * Na | 6.4 | 0.02 |
| MTG | | |
| Tau Braak * Na | 8.0 | 0.01 |
| Sex * Se | 5.6 | 0.03 |
| CG | | |
| Tau Braak * Na | 18.5 | <0.001 |
| Tau Braak * Fe | 18.0 | <0.001 |
| Sex * Se | 10.9 | 0.006 |
| MCX | | |
| PMD * Fe | 6.3 | 0.02 |
| Tau Braak* Na | 6.7 | 0.02 |
| CB | | |
| Tau Braak * Na | 10.1 | 0.005 |
| Sex * Mg | 6.4 | 0.02 |
| Sex * K | 5.5 | 0.03 |
| PVC | | |
| Age * Se | 9.1 | 0.009 |
| Tau Braak * Cu | 9.3 | 0.009 |
| SN | | |
| Tau Braak * Na | 5.0 | 0.04 |
| Sex * Mn | 6.3 | 0.02 |

**Suppl. Table 4.** MANCOVA analysis of covariate effects on individual metals in different brain regions. Design: Intercept + Age + tau Braak Stage + PMD + Sex. Only statistically significant results shown; p < 0.05 considered significant.

# **Suppl. Figure 3. MANCOVA Graphs—Significant Effects on Individual Metals**
